# Recent Developments of Photodeformable Polymers: From Materials to Applications

**DOI:** 10.34133/research.0242

**Published:** 2023-09-29

**Authors:** Shuting Kong, Hailan Wang, Eethamukkala Ubba, Yuxin Xiao, Tao Yu, Wei Huang

**Affiliations:** ^1^ Frontiers Science Center for Flexible Electronics (FSCFE), Shaanxi Institute of Flexible Electronics (SIFE), Northwestern Polytechnical University, 127 West Youyi Road, Xi'an 710072, China.; ^2^ OMC Research Laboratory, Department of Chemistry, School of Advanced Sciences, VITVellore, Tamilnadu, India.; ^3^ Key Laboratory of Flexible Electronics (KLOFE) & Institute of Advanced Materials (IAM), Nanjing Tech University (NanjingTech), 30 South Puzhu Road, Nanjing 211816, China.; ^4^ State Key Laboratory of Organic Electronics and Information Displays & Institute of Advanced Materials (IAM), Nanjing University of Posts and Telecommunications, 9 Wenyuan Road, Nanjing 210023, China.

## Abstract

Photodeformable polymer materials have a far influence in the fields of flexibility and intelligence. The stimulation energy is converted into mechanical energy through molecular synergy. Among kinds of photodeformable polymer materials, liquid crystalline polymer (LCP) photodeformable materials have been a hot topic in recent years. Chromophores such as azobenzene, α-cyanostilbene, and 9,10-dithiopheneanthracene have been widely used in LCP, which are helpful for designing functional molecules to increase the penetration depth of light to change physical properties. Due to the various applications of photodeformable polymer materials, there are many excellent reports in intelligent field. In this review, we have systematized LCP containing azobenzene into 3 categories depending on the degree of crosslinking liquid crystalline elastomers, liquid crystalline networks, and linear LCPs. Other structural, typical polymer materials and their applications are discussed. Current issues faced and future directions to be developed for photodeformable polymer materials are also summarized.

## Introduction

Stimuli-responsive polymer materials, which respond to external stimuli by undergoing prespecified changes, have aroused substantial attention over the last several decades [1]. Such stimuli-responsive polymer materials have the development of far-reaching applications, for instance, chemosensing, medicine transportation, and soft robotics [2–4]. Through external stimuli such as light, heat, humidity, electricity, and magnetic fields, stimuli-responsive polymer materials show obvious changes in physical and chemical properties [5–9]. As an important stimulation source, light possesses unique characteristics of nondirect contact, good temporal and spatial tuning, and a wide stimulation range to drive polymer materials. All stimuli-responsive polymer materials capable of light-responsive shape transformations can be defined as photodeformable polymer materials.

Photodeformable polymer materials can covert shape from original forms to other forms (e.g., twisting, bending, and crawling) by light incentive [1]. Photodeformable polymer materials have many principal advantages such as high-precision control, multifunctionality, and reversibility, which make the system one of the trendiest topics. On the contrary, for molecular crystals, photoinduced deformation is also common due to their advantages of simple synthesis, fast response, and high deformation amplitude [10]. However, molecular crystals are considered brittle, while photodeformable polymer materials are flexible. Therefore, photodeformable polymers are usually applied in the fields of actuators, flexible sensors, and self-healing devices. The several mechanisms of polymers have been reported to date, including photothermal, phase transition, and photoisomerization. Although the shape of polymers changes before and after irradiation on the macroscopic scale, the mechanism is different. For example, the mechanism of photothermal effect is based on transferring heat to mechanical work, which shows the potential in the fields of artificial muscle and encryption storage [11,12]. Meanwhile, the mechanism of phase transition primarily involves the transformation of ordered liquid crystalline (LC) from homogeneous phase to isotropic phase, which can expand the range of applications [13,14]. Photoisomerization is a photochemical process in which conformational changes or chemical bond rearrangements occur within the molecule, ultimately resulting in the formation of isomers [15]. Generally, the existence of photo-responsive groups has a close connection to photoisomerization, which is important in participating in the process of photoinduced deformation. Organic photo-responsive groups, such as azobenzene, stilbene, transition metal complexes, and their derivatives, not only can be doped into the polymer materials to expand the competence of materials but also take part in the preparation processes as mesogenic units and molecular switches to make materials photosensitive [16].

With mechanisms and photo-responsive groups being presented progressively, many photodeformable polymer materials have been developed and applied. At present, light deformation has been realized in different categories of polymers, including liquid crystalline polymer materials (LCPs), hydrogels, shape memory composite (SMC) polymer materials, and carbon-based materials. For example, the gradient LCP actuators are intelligent, such as shape memory and complex programmable deformation [17]. The hydrogels and SMC polymers have their own features, which can be applied in microscale machines and multistimuli response [18,19]. Carbon-based materials possess superior light-to-work conversion efficiency so that the photothermal actuators exhibit tremendous potential for the autonomous systems [20]. Among these different kinds of photodeformable polymers, LCPs are widely studied due to the characteristics of combining the LC and polymers, rapid response, wide-range deformation, and reversibility, which over than others for the fabrication of actuators [21]. However, many photodeformable polymer materials with newly designed and rational molecular structures were reported with fuzzy structure–property relationship and low-innovation functional applications. In 2019, light-responsive shape-changing properties of LCPs, hydrogels, and shape memory polymers were systematically summarized by Stoychev et al. [22]*.* Recently, a review regarding the construction of photocontrollable LCPs containing photosensitive organic dyes and/or inorganic nanocomponents has been reported by Huang et al. [23].

**Fig. 1.**
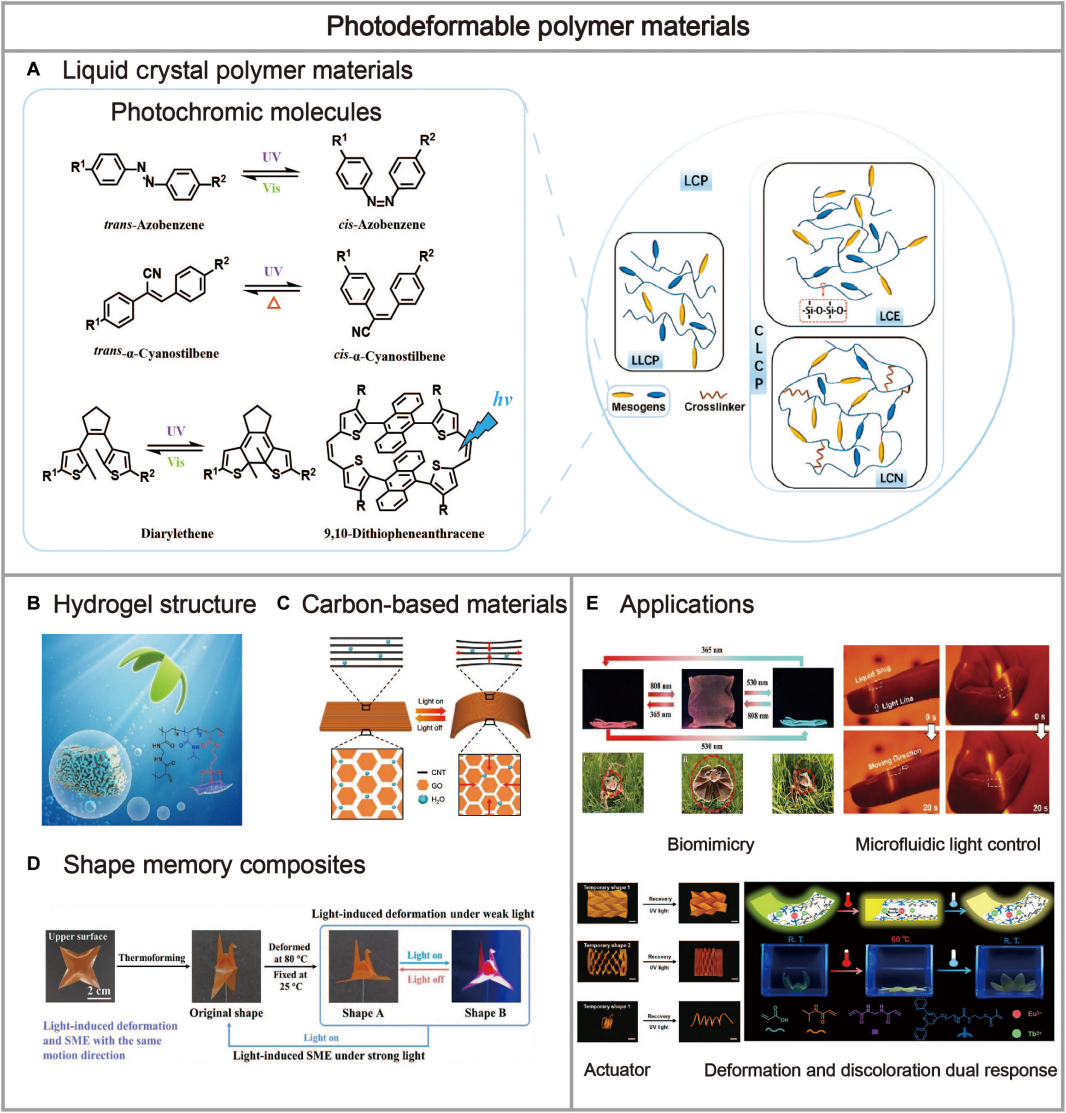
Schematic illustration of photodeformable polymer materials. (A) Schematic of liquid crystalline polymer (LCP) classification. Some representative photochromic chromophores. Reproduced from [14] with permission from the American Chemical Society. (B) Photodeformable polymer materials of hydrogel structure. Reproduced from [18] with permission from Wiley-VCH. (C) Photodeformable polymer materials of carbon-based materials. Reproduced from [68] with permission from Elsevier. (D) Photodeformable polymer materials of shape memory composites. Reproduced from [67] with permission from the American Chemical Society. (E) Applications of photodeformable polymer materials, which include biomimicry, microfluidic light control, actuator, deformation, and discoloration dual response. Reproduced from [59] with permission from Wiley-VCH. Reproduced from [45] with permission from Wiley-VCH. Reproduced from [42] with permission from Wiley-VCH. Reproduced from [82] with permission from Elsevier.

The distinctive properties of materials and their future development led us to analyze reports on such materials. In this review, we summarize the materials based on their types, structures, and properties into 4 categories: LCPs, hydrogels, SMC polymer materials, and carbon-based materials. A series of studies on various photodeformable polymer materials are presented, and the related recent advances in photodeformable behavior and potential applications are also discussed. The strategies and performances of these photodeformable polymers are summarized in Table. By providing the development as well as the cutting edge of photodeformable polymer materials (Fig. 1), we aim to introduce the relationship between the structures and properties of materials and their deformable behaviors and try to identify the following questions: (a) What are the specific factors that affect photodeformation and how do these factors come to affect deformation? (b) How can the strength of the photodeformable behavior be altered and the mechanical properties improved independently of the environment? (c) For what types of applications can these materials be used? With this framework, we conclude by highlighting the current challenges in the field and summarizing the dilemmas and directions in which we should work.

**Table.** Various photodeformable actuation strategies and their performances.

	Name	Main materials	Structure	Size of typical devices	Actuation	Performance(bending angle or curvature or extent of isomerization)	Light source/intensity	Response time	Reference
1	azo-LCEs	RM82/2Azo/*n*-BA/I-874	Monolithic film	5 × 5 mm^2^	Deforming locally	54%	532 nm light, UV light, 50 mW cm^−2^	10 min (532 nm), 5 s (UV)	[26]
2	BLCE512	PMHS/MBB/DR1	Monodomain film	4.0 × 2.0 × 1.5 cm^3^	Bending	115°	520 nm light, 47 mW cm^−2^	23 s	[27]
3	BLCE796	PMHS/MBB/YHD796	Monodomain film	4.0 × 2.0 × 1.5 cm^3^	Bending	111°	808 nm light, 0.18 W cm^−2^	33 s	[27]
4	BLCE1002	PMHS/MBB/Dye1002	Monodomain film	4.0 × 2 .0 × 1.5 cm^3^	Bending	103°	980 nm light, 0.19 W cm^−2^	26 s	[27]
5	LCE fiber	AuNPs/PMMA	Gradient structure	700–800 μm	Bending	13°	UV light, 230 mW cm^−2^	30 s	[28]
6	V-LCE	epoxy oligomer/EAZO/3,6-dioxaoctane-1,8-dithiol mixture	Topological network film	/	Stretching	120 °C	UV light, 160 mW cm^−2^	1 min	[30]
7	SWLFA	GO	single phase with asymmetrical structure	6 × 6 cm^2^	Twisting, coiling, contracting, and shortening	3.3 cm^2^ (1,090%)	808 nm light, 2 W cm^−2^	/	[31]
8	PMP-LC81	LC monomers/A6zA6/A6z2	Higher order state	5 × 1 mm^2^	Bending	2 cm	457 nm light, 7 W cm^−2^	1 s	[32]
9	LCN strip	LC monomers/DR1	Humidity gradient structure	20 mm × 3 mm × 20 μm	Opening	/	450 nm light, 160 mW cm^−2^	1.8 s	[33]
10	LCN/PET	Anisotropic film	Bilayer	20 × 3 × 0.016 mm^3^	Bending, contracting, twisting	Over 5 mm s^−1^	455 nm light, 300 mW cm^−2^; UV light, 170 mW cm^−2^	1 s	[34]
11	Swimmers	Kapton/HDI/A_4_OH/M_6_AzOC_2_	Bilayer	8.0 × 8.0 mm^2^	Rhythmic oscillation	1 cm	UV light, 150 mW cm^−2^	1 s	[35]
12	Poly(azobenzene)s	A6A/1,6-hexane dithiol	Semicrystalline	50 μm	Bending	/	385 nm light, UV light, 100 mW cm^−2^	1 min	[36]
13	AZO-LCDANs	BM/furan/AZO	Bilayer	/	Crawling, rolling	6.8 cm	550 nm light, UV light	21 s	[37]
14	AZOIOs (polymer)	PVA/LC mixture	Bilayer	5 mm × 1 mm × 40 μm	Bending	187.6°	550 nm light, 375 nm light,65 mW cm^−2^	4 s	[38]
15	AZOIOs (PC)	PVA/LC mixture	Bilayer	5 mm × 1 mm × 40 μm	Bending	808.8°	550 nm light, 375 nm light,65 mW cm^−2^	14 s	[38]
16	PAA-DR1/PEO	DR1/PAA/PEO	Complex fibers	0.26 mm	Contracting, bending, waving, curling	20%	UV light, 10 W cm^−2^	/	[39]
17	PAA-Aazo/PEO	Aazo/PAA/PEO	Complex fibers	0.26 mm	Contracting, bending, waving, curling	25%	UV light, 10 W cm^−2^	/	[39]
18	AZO-DAN	AZO/furan/BMI	Ordered state film	20 × 5 × 0.03 mm^3^	Bending	/	530 nm light, 30 mW cm^−2^; UV light, 40 mW cm^−2^	/	[41]
19	PU-1	DH6AB/HD/MPD/MDI	Anisotropic film	5 mm × 2 mm × 16 μm	Bending	40°	>540 nm light, 40 mW cm^−2^; UV light, 10 mW cm^−2^	40 s	[43]
20	PFM	PABBP/EVA	Bilayer	1.5 m × 125 μm (thick)	Climbing	6.8 mm	470 nm line light, 120 mW cm^−2^	17 s	[44]
21	TMA	C11AB6	Highly ordered and nano-scaled lamellar	0.5 mm × 8 μm (thick)	Climbing	2 mm	470 nm light, 125 mW cm^−2^	20 s	[45]
22	PC-AB6	C-AB6	Spontaneous orientation film	10 mm × 1.57 mm × 20 μm	Bending	92°	530 nm light, 140 mW cm^−2^, UV light, 80 mW cm^−2^	20 s (530 nm), 6 s (UV)	[46]
23	PC-3AB6	C-3AB6	Spontaneous orientation film	10 mm × 1.57 mm × 20 μm	Bending	50°	530 nm light, 140 mW cm^−2^, UV light, 80 mW cm^−2^	12 s (530 nm), 2.5 s (UV)	[46]
24	PC-7AB6	C-7AB6	Spontaneous orientation film	10 mm × 1.57 mm × 20 μm	Bending	65°	530 nm light, 140 mW cm^−2^, UV light, 80 mW cm^−2^	6 s (530 nm), 2 s (UV)	[46]
25	PNb-AB4	PEG200/DMAP/PEO	Nematic phase film	10 mm × 2 mm × 20 μm	Bending	60°	530 nm light, UV light, 80 mW cm^−2^	3 s	[47]
26	PNb-6AB4	PEG200/DMAP/PEO	Nematic phase film	10 mm × 2 mm × 20 μm	Bending	72°	530 nm light, UV light, 80 mW cm^−2^	2 s	[47]
27	LLCP fiber	Hydroquinone/4-hexyloxybenzoic acid/DMAP	Isotropic phase	1.5 cm	Contracting, stretching	81%	470 nm light, 100 mW cm^−2^	53 s	[48]
28	PC11AE6	4-hexyloxybenzoic acid/DMAP/EDC/C11AE6	Anisotropic structure	5 mm × 1 mm × 10 μm (film), 40 μm (fiber)	Bending	68° (film), 110° (fiber)	Visible light, 50 mW cm^−2^, UV light, 40 mW cm^−2^	30 s (530 nm), 60 s (UV, film), 30 s (UV, fiber)	[49]
29	Liquid slugs	PMMA	Asymmetric deformation	20 μm	Moving	11 μm	470 nm blue light, 80 mW cm^−2^	1 s	[50]
30	Pt-BA2DA-PVDF	BA2DA/Pt	Single-crystal structure	-	Bending	1 cm	440 nm light	10 s	[51]
30	P1-100k	AZO/PMDETA/MBPA	Polydomain film	7 mm × 2 mm × 10 μm	Bending	62°	470 nm light, 9 mW cm^−2^, UV light, 51 mW cm^−2^	50 s (470 nm), 10 min (UV)	[52]
31	LCE-PM10PVPC4	M10PVPC4/ME6UPy	Double-layer smectic phase	-	Bending	113°	UV light	9 s	[54]
32	LCE-10CS	M*mCS*12/ME6UPy	Monodomain structure	1 ± 0.1 mm × 20 ± 0.1 mm	Bending	130°	UV light, 120 mW cm^−2^	20 s	[55]
33	P4VP(Z-TCS)_1.0_	Z-TCS/P4VP	Uneven contraction fiber	1 mm × 20 mm	Bending	90°	UV light, 80 mW cm^−2^	8 s	[3]
34	DAE-LCN	Diarylethene/IRG819	Anisotropic film	3 × 0.5 × 0.02 mm^3^	Bending	11°	550 nm light, 285 mW cm^−2^, UV light, 50 mW cm^−2^	2 s	[57]
35	8	Di(3-hexylthienyl)-9,10-anthracene	Overall strained structure	1,000 × 100 × 5 μm^3^	Bending	16.3°	445 nm, 0.13 W cm^−2^	10 s	[58]
36	LCE0	YHD796	Monodomain film	/	Crawling	1.1 cm	808 nm light, 0.19 W cm^−2^, UV light	/	[59]
37	LCE1	TPE/SP	Polydomain film	/	Crawling	1.1 cm	808 nm light, 0.19 W cm^−2^, UV light	/	[59]
38	LCE2	YHD796/TPE/SP	Hybrid local alignment	/	Complex shape-morphing of 3D	/	530 nm light, 808 nm light, UV light	/	[59]
39	azo-PI (air)	6FDA/DAC3AB	Anisotropic film	5 mm × 1 mm × 20 μm	Bending	112°	530 nm light, 80 mW cm^−2^, UV light, 70 mW cm^−2^	10 s (530 nm), 8 s (UV)	[60]
40	azo-PI (water)	6FDA/DAC3AB	Anisotropic film	5 mm × 1 mm × 20 μm	Bending	45°	530 nm light, 80 mW cm^−2^, UV light, 70 mW cm^−2^	15 s (530 nm), 8 s (UV)	[60]
41	azo-PI (80 °C silicone oil)	6FDA/DAC3AB	Anisotropic film	5 mm × 1 mm × 20 μm	Bending	45°	530 nm light, 80 mW cm^−2^, UV light, 70 mW cm^−2^	25 s (530 nm), 12 s (UV)	[60]
42	0.5-1FPP	P(NIPAM-ABP)/Fe_3_O_4_/PAN	Anisotropic bilayer	76 μm	Bending	720°	NIR-II light, 2.65 W cm^−2^	107.2 s	[65]
43	2.5-1FPP	P(NIPAM-ABP)/Fe_3_O_4_/PAN	Anisotropic bilayer	76 μm	Bending	720°	NIR-II light, 2.65 W cm^−2^	9.7 s	[65]
44	CUP/PT	CUP NSs/PT	Bilayer	40 × 20 × 0.1 mm^3^	Folding	96%	1,064 nm light, 0.4 W cm^−2^	120 s	[66]
45	HNAN-NO_2_/P(VDF-TrFE)	HNAN-NO_2_/P(VDF-TrFE)	Bilayer	30 mm × 3 mm × 60 μm	Expanding	4.1 mm	648 nm (red) light, 50 mW cm^−2^	/	[67]
46	HNAN/P(VDF-TrFE)	HNAN/P(VDF-TrFE)	Bilayer	30 mm × 3 mm × 60 μm	Expanding	2.6 mm	532 nm (green) light, 50 mW cm^−2^	/	[67]
47	HNAN-CH_2_/P(VDF-TrFE)	HNAN- CH_2_/P(VDF-TrFE)	Bilayer	30 mm × 3 mm × 60 μm	Contracting	2 mm	450 nm light (blue), 50 mW cm^−2^	/	[67]
48	GO layer	GO/CNT	Bilayer	23 mm × 3.5 mm	Jumping	8 mm	NIR light	0.14 s	[68]
49	GO-PNIPAM	GO/PBI-HPEI	Anisotropic bilayer	/	Bending	720°	532 nm light (green)	600 s	[69]
50	AZ-CLCPs	LC monomer/AIBN	Random copolymers	10 mm × 0.3 mm	Bending	/	UV light, 192 mW cm^−2^	60 s (80 °C)	[73]
51	PU-T	NIR-absorbing diol 3/HDI/PTMEG/TEA	Anisotropic film	0.45 mm	Stretching, shrinking	200%	808 nm light, 2 W cm^−2^	60 s	[74]
52	PVDF-CB/PEA/PAM	PAM/PVDF/CB	Anisotropic film	15 mm × 2 mm × 150 μm	Jumping	199°	NIR light, 250 mW cm^−2^	0.56 s	[75]

RM82, 1,4-bis-[4-(6-acryloyloxyhexyloxy)benzoyloxy]-2-methylbenzene; 2Azo, 4,4′-bis[6-(acryloyloxy)hexyloxy]azobenzene; *n*-BA, *n*-butylamine; I-784, Irgacure 784; PHMS, poly(methylhydrosiloxane); MBB, mesogenic monomer 4-but-3-enyloxy-benzoic acid 4-methoxy-phenyl ester; YHD, photothermal dye; DR1, Disperse Red 1; PMMA, polymethyl methacrylate; PDMS, polydimethylsiloxane; IPDI, isophorone diisocyanate; SS, 4,4′-dithiodianiline; BNB, 4,4′-bis(hydroxymethyl)-2,2′-bipyridine; EAZO, bis(oxiran-2-ylmethyl) 4,4′-(diazene-1,2-diyl) (E)-dibenzoate; GO, graphene oxide; NMP, *N*-methylpyrrolidinone; SP, spiropyran; 6FDA, 1,1,1,3,3,3-hexafluoro-2,2-bis(4-phthalic anhydride)-propane; DAC3AB, azo-containing diamine monomer; FPP, Fe_3_O_4_/P(NIPAM-ABP)/PAN; DMAc, *N*,*N*-dimethylacetamide; PAA, poly(amic acid) oligomers; AA, acrylate; AIEM, AIE molecules; BM, bismaleimide; PVA, polyvinyl alcohol; BMI, 1,6-bis(maleimido)hexane; DH6AB, 4,4′-bis(6-hydroxyhexyloxy)azobenzene; HD, 1,6-hexanediol; MPD, 3-methyl-1,5-pentnanediol; MDI, methylenediphenyl 4,4′-diisocyanate; EVA, ethylene-vinyl acetate copolymer; PEG200, polyethylene glycol; DMAP, 4-dimethylaminopyridine; PEO, methoxypolyethylene glycols; EDC, 1-(3-dimethylaminopropyl)-3-ethylcarbodiimide hydrochloride; PMDETA, *N*,*N*,*N*′,*N*′′,*N*′′-pentamethyldiethylenetriamine; MBPA, methyl-α-bromophenylacetate; M*mCS*12, α-cyanostilbene monomer; ME6UPy, hydrogen bond monomer; P4VP, hydrogen-bonded to poly(4-vinyl pyridine); DAAB, 4,4′-diaminoazobenzene; HEMA, hydroxyethyl methylacrylate; NVP, *N*-vinyl-2-pyrrolidone; CUP NSs, CaSiCu_4_O_10_ nanosheets; PT, poly (d,l-lactide-co-trimethylene carbonate); HNAN, 2-hydroxynaphthylidene-1′-naphthylamine; PBI-HPEI, perylene bisimide-functionalized hyperbranched polyethylenimine; AIBN, 2,2-azobis(isobutyronitrile); HDI, 3,4,hexamethylene diisocyanate; PTMEG, polytetramethylene ether glycol; TEA, triethanolamine; PAM, polyacrylamide; PVDF, poly(vinylidene fluoride); CB, carbon black; PNIPAAm, poly (*N*-isopropylacrylamide); PA6ACA, poly (acryloyl-6-aminocaproic acid).

## Photodeformable materials based on LCPs

### Crosslinked LCPs

Crosslinked LCPs (CLCPs) are stimuli-responsive materials. Common stimulus sources include light, electricity, heat, and magnetic fields. Based on the degree of backbone flexibility, crosslinking, and glass transition temperature (*T*_g_), CLCPs can be divided into liquid crystalline elastomers (LCEs) and liquid crystalline networks (LCNs) [24,25].

Due to the highly flexible backbone, LCEs exhibit both the properties of LC materials and elastomers. LCEs consisted of crosslinked LC side-chain and/or main-chain mesogenic units, but the polymer backbones have flexibility and a low overall crosslink density. These materials possess the ability to undergo large mechanical deformations while maintaining their LC order and can be programmed into different shapes by external stimuli. Moreover, LCNs are formed by a higher degree of crosslinking, resulting in a more rigid polymer network. This increased crosslinking restricts the backbone flexibility, leading to enhanced mechanical strength and stability. Compared to LCEs, LCNs typically exhibit a higher *T*_g_ and achieve a transition of glassy state with higher temperatures.

The division of CLCPs into LCEs and LCNs allows for a classification that considers the varying degrees of backbone flexibility, crosslinking density, and thermal properties, enabling researchers to tailor the material’s characteristics for specific applications in areas such as soft robotics, actuators, and smart materials. It will be summarized by introducing their background, manufacturing methods, advantages, and latest work progress.

#### Liquid crystalline elastomers

LCEs are a type of CLCPs. It is one of the soft actuating materials with unique macroscopic reversibility and complex flexible deformation. LCEs are a class of soft, functional materials that can reversibly change shape in response to environmental stimuli. These lightly crosslinked polymers combine the orientational order of LCs with the entropic elasticity of elastomers. Shape change is the result of a change in the ordering parameter. This type of shape change can be programmed into the polymer network by controlling the molecular orientation in the network before crosslinking.

**Fig. 2.**
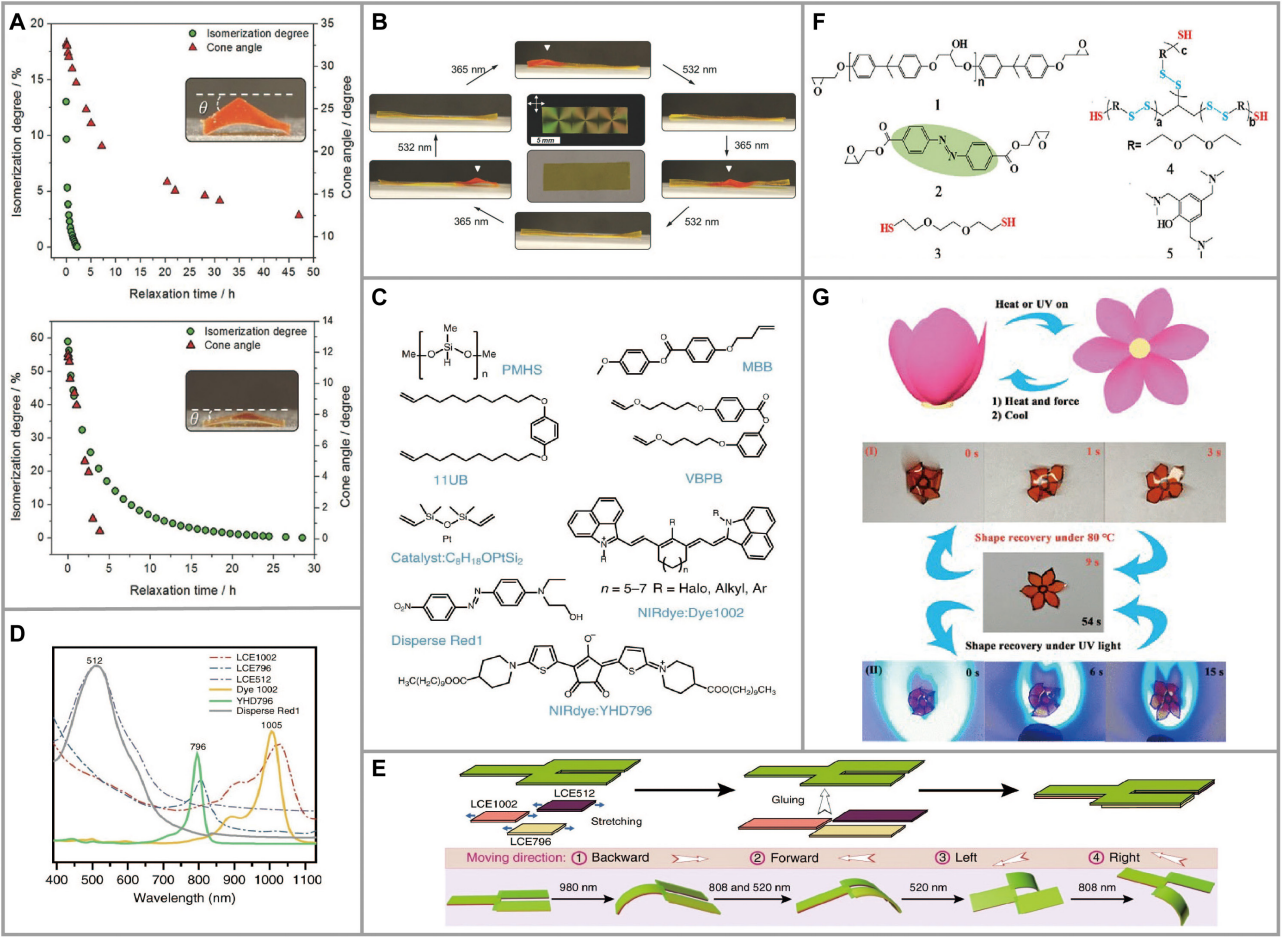
(A) Comparison of the *cis-trans* thermal relaxation of materials prepared from compositions 3 and 5 and the mechanical recovery of the flat state. (B) The UV irradiation actuates the conical domes in the film locally. Subsequent exposure to 532-nm light restores the film to the original flat state. Reproduced from [26] with permission from Wiley-VCH. (C) Chemical components required in this LCE system. (D) UV–Vis–IR absorption spectra of YHD796, Dye1002, and Disperse Red 1 in dichloromethane, and the corresponding LCE796, LCE1002, and LCE512 films. (E) Photo-modulated multidirectional bilayered LCE walker. Reproduced from [27] with permission from Springer Nature. (F) Chemical structures of the materials used to prepare V-LCE. (G) Schematic of the heat- or light-driven behavior of the bionic flower-shape actuator under UV light irradiation (160 mW cm^–2^). Reproduced from [30] with permission from the American Chemical Society.

A reversible shape transition from two-dimensional (2D) to 3D can be achieved under light illumination with azobenzene (azo-LCEs) as the main chain was reported [26]. The magnitude of this deformation is determined by the azobenzene concentration and the crosslinking density. The crosslinking density was adjusted by the ratio of acrylate to *n*-butylamine, and a slightly higher concentration of *n*-butylamine increases the concentration. Optimal photoresponse behavior can be obtained when azo-LCEs reached the lowest crosslinking point density. The group used the cone top and time shift to determine whether the crosslinking density was effective for photodeformation. The results showed that higher crosslinking density leads to faster cone recovery (Fig. 2A and B).

In 2019, Yang et al. [27] established LCE systems based on polymethyl-hydrosiloxane and synthesized 3 LCEs responsive to different wavelengths (520/808/980 nm) by each material (Fig. 2C and D). Under the irradiation of infrared light at 3 different wavelengths, namely, BLCE512, BLCE796, and BLCE1002, the films exhibited induced behavior as they bent upward. Remarkably, all 3 films bent more than 103° within 30 s. The layered structure of LCE materials made from the above 3 films can integrate multiple independent and undisturbed photothermal conversion systems that respond to different wavelengths (Fig. 2E).

Moreover, the first case of photothermal deformation with LCE fibers doped with AuNPs in the presence of waveguides was demonstrated [28]. Unlike other photodeformation mechanisms reported in the literatures, this is a deformation controlled by the photoconductivity of the gold salts. Under the light, the fibers bent toward the direction in which the AuNPs were attached. The surface temperature to which the AuNPs were drop-added increases, and the shrinkage along the main tube decreased as the temperature decreases, resulting in bending. This fiber could also achieve bending in multiple directions to precisely control the structure of the desired pattern.

In 2020, a simple fabrication method was developed to program the main chain of LCE by using a 2-step photocrosslinking process to create a 3D structure. First, linear monomer of dipropionate and *n*-butylamine extended the chain by Michael addition reaction to obtain LC oligomers [29]. Then, 2 photocrosslinking processes were performed: the first photocrosslinking partially cured the LC oligomer and the transparent sample becomes opaque to form a multidomain LCE, and the second photocrosslinking fixed it for 30 min at room temperature to obtain a fully crosslinked multidomain. The domain LCE locked the alignment in the LCE and mechanically programmed the desired shape. In addition to shape programming, the relationship between driving strain and elongation of multidomain LCE was explored in this work. The thermally actuated strain of single-domain LCE was strongly dependent on the initial degree of stretchability of multidomain LCE. As shown by laser scanning microscopy, the interior region of the fixed shape LCE micro-pillar under ultraviolet (UV) irradiation exhibited a change in pillar height when it was reversibly subjected to heat. Without the application of photodeformation, we can understand the effect of UV light on crosslinking activity through this article.

In 2022, Zhang et al. [30] introduced azobenzene units and dynamic disulfide bonds in LCE to design and obtain a dynamically actually 3D structured actuator with self-healing and biomimetic properties. The vitrimer LCE (V-LCE) showed excellent functions in absorbing, transferring, and releasing materials with the help of azobenzene molecules and disulfide bonds with epoxy resin and thiol compounds in the actuator main chain curing reaction. A schematic diagram of the structure required for the preparation of V-LCE materials is shown, and the self-healing via disulfide bond exchanged reaction (Fig. 2F). The shape-changeable actuator exhibited blooming and contracting behavior under external stimuli (Fig. 2G). This lays a solid foundation for the development of intelligent bionic prosthetic devices.

LCE has designed fiber optic actuators with excellent performances [31]. This actuator could undergo thousands of light deformation reversible behaviors without any significant fatigue effect. In addition, this bio-inspired actuator had a high degree of freedom and can achieve complex deformations. Actuator fabrication involved graphene, which effectively responded to near-infrared (NIR) spectroscopy with a light intensity of 3 W cm^−2^, could achieve a contraction rate of 1,750% and a fast drive rate of 258% s^−1^, proving its excellent actuation performance.

Three mixtures of liquid crystals comprised entirely of azobenzene molecules were reported, with a focus on investigating influence of the ratio of linear monomer and crosslinker on their properties [32]. Experimental results demonstrated that the photodeformation behavior of actinic material with L:C = 8:1 works best in parallel polarized light at 447 nm. This was attributed to a higher degree of crosslinking at this ratio, whereas both 1:8 and 1:1 resulted in a decrease in the strength and stiffness of the fibers. Photodeformation behavior could be restored by illuminating vertically polarized light. The result could be further applied to thin films.

#### Liquid crystalline networks

Apart from LCEs, LCNs are also one of the most promising classes of stimuli-responsive materials. LCNs are polymer networks with tunable optical, mechanical, and electrical properties that have found application in widely commercialized LC display technologies. However, as the field matures, the use of LCNs as stimuli-responsive materials for soft actuators and sensors has become a focus of interest.

**Fig. 3.**
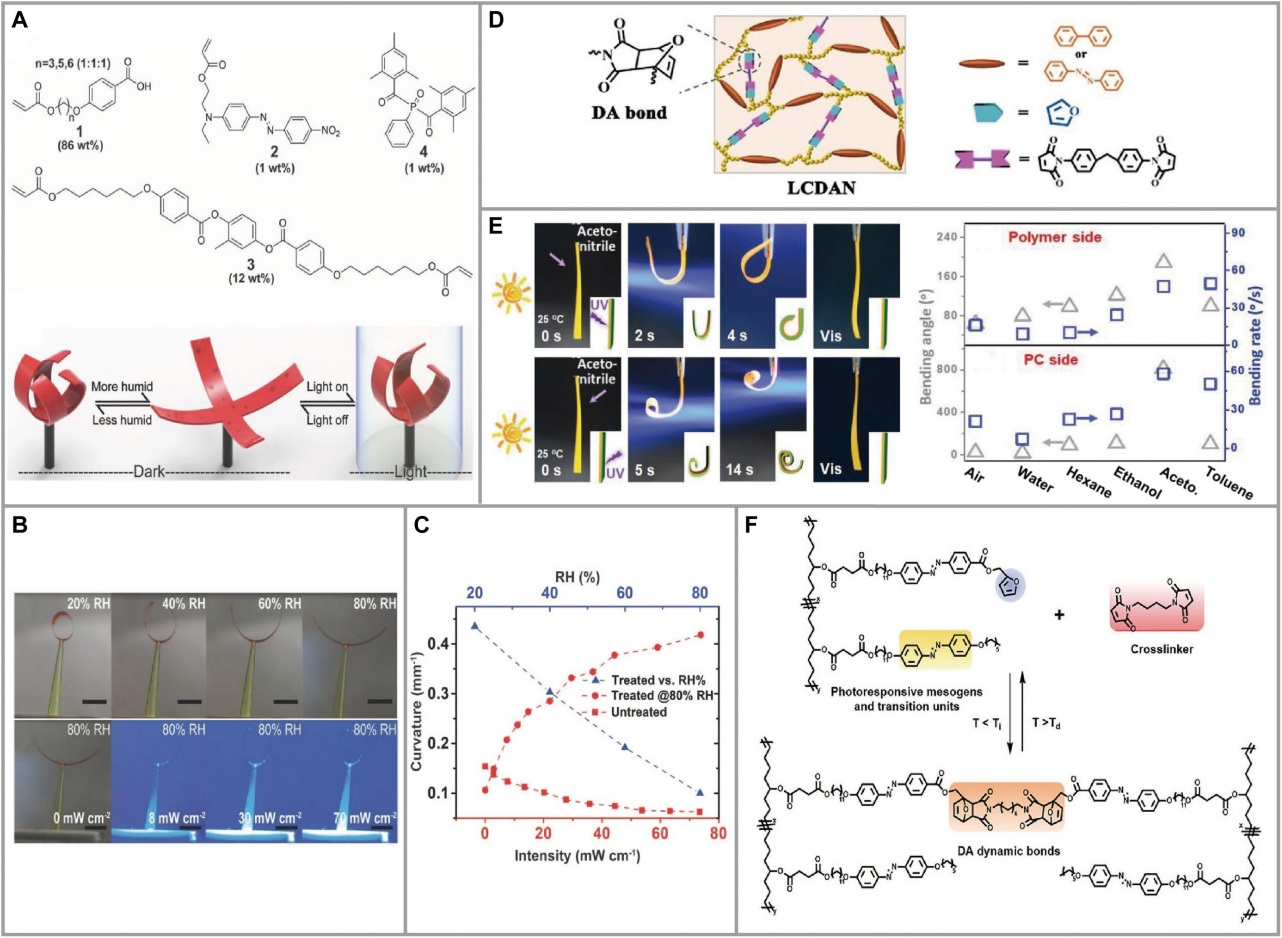
(A) Schematic representation of the nocturnal actuator: humidity level controlled the opening and closing of the flower and composition of the LC mixture. (B) Curvature changes with variation in RH, and effect of light intensity on curvature of LCN strip held at 80% RH (room temperature). (C) Curvature changes in response to increasing RH (blue triangles), and the photo-induced curvature change of treated (red circles) and untreated (red squares) LCN strips held at 80% RH in response to increasing light intensity. Reproduced from [33] with permission from Wiley-VCH. (D) Chemical structures of the “self-lockable” actuators. Reproduced from [37] with permission from Wiley-VCH. (E) Enhanced photoresponsive actuation of the Janus AZOIO film in acetonitrile at 25 °C and different solvents and air upon UV irradiation. Reproduced from [38] with permission from Wiley-VCH. (F) Chemical structures of Diels–Alder network (AZO-DAN) and photographs to show the light-induced shape memory performance. Reproduced from [41] with permission from Wiley-VCH.

Schenning et al. [33] demonstrated a floral LCN photoactivator doubly coordinated by light and moisture (Fig. 3A). This LCN actuator was produced by polymerization of azobenzene derivatives dispersed in red 1 acrylate and acrylate-based carboxylic acid monomers of various chain lengths through hydrogen bonding at 80 °C. Alkali action makes the film sensitive to moisture. Compared to the traditional photo-thermal reaction, the photo-hygroscopic reaction is more rapid. The blooming and shrinking of flowers in dark conditions depend on the amount of moisture. An increase in relative humidity (RH) led to a linear decrease in the curve. Also, when RH was 80%, the increase in light irradiance reduced the response time to 1.8 s (Fig. 3B and C). In 2020, they made a thermoplastic photo-responsive LCN that could be programmed with arbitrary shapes [34]. A 4 μm/12 μm LCN/polyethylene terephthalate film could be easily bent in UV to achieve the same bending speed as a single layer of LCN films thicker than 20 μm. Specifically, removal of the UV film did not restore the original shape, but blue light exposure restored the deformed film to its programmed state. The group programmed the film with a sharp accordion shape that allows for folding and unfolding movements. This actuator is versatile and easy to manufacture.

An LCN/Kapton composite film by controlling the height of light could be used at room temperature to simulate the swimming behavior of dolphins [35]. This LCN layer containing azobenzene has excellent photoresponse performance. The bilayer composite film bent toward the Kapton within 1 s and returned to its original shape within 2 s when the UV light is removed. Unlike what has been reported in other literature, here, this group did not use visible light and heat to restore the film to its original shape. Moreover, the degree of photodeformation was positively and linearly related to light intensity. Due to the swimming behavior of the bionic dolphin, the motion of the photodeformable film can be explored in a mixture of ethanol and water and its orientation is very simple.

In 2020, a stepwise semicrystalline main-chain azo polymer [p(A6)] using the Michael addition polymerization of azobenzenedipropionate (A6A) with 1,6-hexanedithiol in a 1.025:1 molar ratio was synthesized. The crystallization of these materials was controlled by isomerization, which could be reversibly melted and recrystallized after nearly athermal photoisomerization, confirming this reversibility of crystallization with differential scanning calorimetry (DSC) [36]. Polymer fibers were drawn with a melt containing 2.5 wt% photoinitiators, and 405 nm light crosslinked the acrylate ends at 80 °C to make aligned fibers. A reversible photoactuator capable of producing mechanical work through light fusion can be designed. In this work, the change in radius of curvature of *D* = 50 μm fibers during illumination was monitored to study the driving properties of the material, with a minimum radius of curvature at room temperature and a sharp increase in curvature as the temperature increases and the semicrystalline *trans* p(A6) melts. There was a reversible behavior at 60 °C with continuous UV and green light irradiation for 1 min. This approach to photoreactive materials offers new opportunities in the field of photostretchable devices.

Zhao et al. [37] fabricated 2 self-locking LC Diels–Alder dynamic network (LCDAN) actuators. Both actuators contained several furan side groups of Diels–Alder-bonded polyesters. One of the LCDAN actuators was formed by polymerization with an azobenzene (AZO) intermediate, referred to as AZO-LCDANs, while the other used a biphenyl (BP) intermediate and was named BP-LCDANs (Fig. 3D). Any of the above polymers dissolved in tetrahydrofuran with a random number of bismalimide can program LCDANs as self-locking 3D structured actuators at room temperature, and the light-dependent deformation and recyclability of LCDANs were investigated. The design considers that this material requires a phase transition temperature (*T*_LC-iso_) that is prominently below the DA bond dissociation temperature, converting it from order to disorder, so that reversible phase transitions can be performed without breaking the DA network. Based on this, an arch walker (AZO-LCDANs and UbwPu composite films) and a wheel rotator were programmed by temperature control, both moving well under UV irradiation. The properties of these materials include room temperature programming, alignment meson self-locking, and easy erasure at high temperatures for reprogramming or recovery from solution. These properties make them promising materials in various applications. A simple method was used to prepare a Janus AZO actuator that still had excellent photodeformation behavior in various liquid environments [38]. The actuator was introduced with one side of a polydomain AZO inverse opal structure and the other side of a monodomain AZO polymer. The actuators exhibited significant bending in tetrahydrofuran, chloroform, and dichloromethane because of the different swelling/dissolving abilities of the 2 sides in the solvent and the different directions of film bending in different solvents. The bending angle of the actuator increased from 20.8° (air) to 808.8° at 14 s in acetonitrile (Fig. 3E). Two azobenzene compounds, poly(acrylic acid) oligomers (PAA)-Disperse Red 1 (DR1) and PAA-Aazo, combined with poly(ethylene oxide) (PEO) to form fibers with hydrogen bonds [39]. Both behaved differently under UV. The mechanical properties of the orange PAA-Aazo/PEO fiber were significantly improved due to higher thermal relaxation, while the red PAA-DR1/PEO fiber showed no change. Both stretched fibers contracted rapidly under 25% and 20% light, respectively. This is due to the isomerization of azobenzene creating free volume in the fibers. The strain capacity stored in the stretched fibers is rapidly released, leading to the contraction of the fibers.

By describing the deformation mechanism through free volume theory within the LCN, Slot et al. [40] showed the influence of the arrangement of the LCN on the active dynamic surface. Once a simple polymer network has formed a stable structure, it needs external stimuli to destabilize its molecular arrangement to achieve macroscopic changes. The freedom of regions aligned vertically to the degree of deformation, and the free volume of molecules is more important. Oscillations caused by light, electric fields, and other factors increased the free volume of molecules, resulting in the formation of protrusions on the surface of their films. These protrusions, in turn, led to changes in the mechanical modulus and other properties of the films.

Diels–Alder bonds have attracted much attention due to their mild topology solidification transition temperature, and this dynamic network can be topologically rearranged above the topology-freezing transition temperature to alter the 3D shape reprogramming of LCN. AZO-DAN was obtained from linear LCPs (LLCPs) and the crosslinking agent 1,6-bis(maleimido) hexane (Fig. 3F) [41]. Among them, the furan region of LLCP formed a Diels–Alder network with BMI [1,6-bis(maleimido)hexane], which stabilized the structure of the mesocrystal arrangement under the action of optimal annealing temperature. The *cis*-AZO-DAN in the rubber state is programmable under UV radiation. *Trans*-AZO-DAN in the glassy state corrects the shape. This group used ordered butterflies and disordered flowers and a dual combination of the 2, realized under the irradiation of alternating light sources for the phenomenon of wings flying over flowers. The Diels–Alder reaction was also utilized to prepare surface-aligned images with LCN as the backbone [42]. In the previous description, the Diels–Alder cycloaddition was primarily employed for crosslinking purposes. This group used a one-pot method to prepare the LCN backbone without the action of additives. LCN self-supported films prepared only at 78 °C showed good alignment. Mechanical strengths of 0.5 to 2 GPa and moduli with a 2-fold difference could be achieved. Aligned LCN films prepared by this method pave the way for the subsequent introduction of photochromic groups and applications.

Combining azobenzene molecules with polyurethane (PU) to achieve a hydrogen-bonded physical crosslinking network differs from crosslinking by covalent bonds [43]. In most of the reported articles, the materials studied for physically crosslinked networks are fibers and films, and thus, a breakthrough is to study the photodeformation behavior of thermoplastic PUs. PU-1 materials can change their macroscopic shape mainly by annealing temperature. The *cis*-*trans* conformation of the azobenzene structure under light leads to a change in the orientation of the chain segments, resulting in a macroscopic deformation observable by the human eye.

### Linear LCPs

LLCPs do not require chemical crosslinking to have good mechanical properties as well as LC orientation. Most LCPs are polymerized by chemical crosslinking. However, LLCPs are composed of high molecular weight that use ring-opening metathesis polymerization (ROMP), which breaks through a complex manufacturing process to solve the messy synthesis of LCPs. LLCPs can be processed using traditional melting methods and solution treatments, allowing for efficient fabrication of intricate designs and tailored properties, thus overcoming the challenges associated with the complex synthesis of conventional LCPs.

**Fig. 4.**
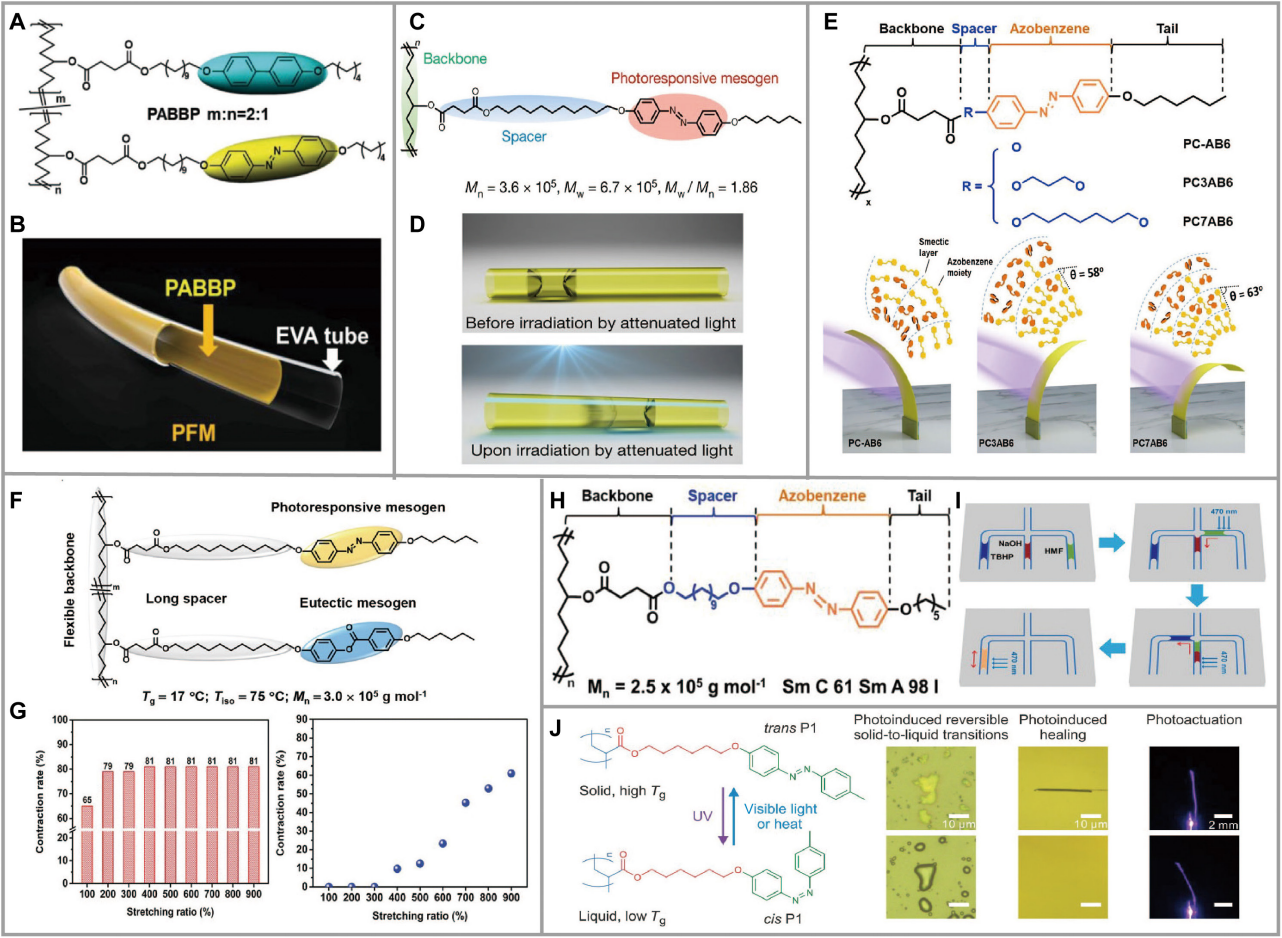
(A) Chemical structure of the PABBP copolymer. (B) Schematic representation of the bilayer structure of the PFM. Reproduced from [44] with permission from Wiley-VCH. (C) Molecular structure of a novel linear liquid crystalline polymer (LLCP). (D) Schematics showing the motion of a slug of fully wetting liquid confined in a TMA driven by photodeformation. Reproduced from [45] with permission from the American Chemical Society. (E) Chemical structures of LLCPs with different spacer lengths, and the photoinduced bending of the LLCP films with different mesogen orientations formed spontaneously after annealing. Reproduced from [46] with permission from Wiley-VCH. (F) Chemical structures of new LLCPs. (G) Light-driven contraction rates versus stretching ratios of the LLCP fibers. Schematic illustration of different stretching ratios on contraction rates of the LLCP fibers with loading. Reproduced from [48] with permission from Wiley-VCH. (H) Chemical structure of the photodeformable LLCP. (I) With 470-nm light, the hydroxymethylfurfural is fused with NaOH and tert-butyl hydrogen peroxide sequentially, and the fused liquid slugs are mixed uniformly. Reproduced from [50] with permission from Wiley-VCH. (J) Chemical structure, photoresponsive properties, entanglement models, and mechanical properties of the azopolymer P1. Reproduced from [52] with permission from Wiley-VCH.

Flexible microtubes are an assembled, optional modular component for microfluidic devices. A 2-layer photocontrollable flexible microtube (PFM) with a commercially available ethylene-vinyl acetate (EVA) layer as a support layer and a 5 wt% dichloromethane copolymer (PABBP) solution coated internally to form a deformed PABBP layer was fabricated (Fig. 4A and B) [44]. This PFM tube can be molded into any shape and can be knotted or suspended from a weight of 200 g without damage. Slugs were induced to move in a controlled direction and climb an 11° slope at 0.4 mm s^−1^ by irradiating one end of an isopropanol bullet confined in microtubes with a 470 nm spot. Light-controlled deformation of the PABBP layer geometry from a cylindrical to a conical region in a PFM tube was observed by super-resolution microscopy, where asymmetric expansion occurred along the long axis of the microtubule, resulting in asymmetric capillary forced driving fluid motion. The article conducts experiments on fluid motion on the finger, and a PFM attached to the index finger changes its trajectory with different hand gestures.

In 2016, the study introduced the first design of a phototropic tubular microactuator (TMA) capable of easily manipulating liquid motion. This TMA exhibited asymmetric deformation when stimulated by 470-nm light, thereby generating uneven capillary forces that drive the liquid forward (Fig. 4C and D) [45]. Previously, TMAs made from CLCPs exhibited poor processability during chemical crosslinking. However, this TMA was prepared using a new type of LLCP, which is more robust. It had a strength of up to 20 MPa and an elastic modulus of 96 ± 19 MPa. These values indicated that, when combined with a well-designed LLCP, TMAs could enhance their mechanical properties. Yu et al. [46] synthesized 3 spacers with different lengths and intermediates with spontaneously oriented photodeformable LCPs to form TMA annealed films by CH_2_CI_2_ solution processing and cutting to length and explored the relationship between different intermediate lengths and phase transition, bending behavior, and fluid transport (Fig. 4E). DSC determined that LLCPs gradually stabilized the LC phase with increasing spacer length. Under UV/visible light irradiation, PC-AB6 films bent toward the light source in 6 s, while PC-3AB6 and PC-7AB6 bent away from the light source direction in 2.5 and 2 s and recovered rapidly under visible light. This was because the anisotropic shrinkage caused by the photoisomerization of PC-AB6 was mainly produced in the intermediate orientation direction on the film surface, the photoisomerization caused the surface swelling of PA-*n*AB6, and the swelling increased with growth of spacer lengths. In the liquid transport of PC-*n*AB6 TMA with an inner diameter of 500 μm, the light intensity gradually decayed from left to right, the isopropanol slug with the uneven concave surface was pushed to the right, the intensity decay was reversed, the slug moved reverse, the length of the intermediate was longer, the angle of inclination was larger, and it moved faster. When the inclination angle was 90°, the coaxial orientation and the speed were at their maximum.

Yu et al. [47] used a simple ROMP to polymerize 2 photosensitive lateral LLCPs with different spacer lengths, polymer SLLCPs, with azobenzene as the side chain, and processed them into thin films by a melt shear-induced orientation method. Flexible spacers were investigated on the photodeformation behavior and deformation behavior of the films. The molecular weight and polymer dispersion indices of SLLCPs show that the polymers synthesized in this work have an order of magnitude higher molecular weight than other LCPs and that higher molecular weight improves the mechanical processing properties of materials. The higher *T*_g_ of PNb-AB4 than PNb6AB4 is due to the decoupling and reduced hindrance to the movement of the main side chains by the flexible spacers of PNb6AB4. The high *T*_g_ of PNb-AB4 and PNb6AB4 reached 60° and 72° in 3 and 2 s under UV light irradiation, respectively. Due to the low coupling effect between the main chain and the azo phenyl group, films with long spacers bent faster and at larger angles. Moreover, the films bent under visible light, indicating that the SLLCPs have good shape stability.

Combining azobenzene and benzoate to form LLCP fiber based on the principle of shape memory effect and photochemical phase change and introducing benzoate as a eutectic intermediate is beneficial to activate chain mobility to a greater extent (Fig. 4F) [48]. The fibers were stretched above *T*_LC-iso_ to self-assemble the azobenzene and benzoate intermediates in the side chains into a sheet-like structure, and after cooling to room temperature, the fibers kept their stretched shape without a rebound. The highly ordered structure of the LC phase in this process inhibits the skeleton movement and locks the strain energy in the fiber. Under the light, the azobenzene LC lamellar structure was disrupted by isomerization, allowing the release of prestored strain energy, leading to shrinkage of the fiber, which is 81% (without applied load, stretched up to 400%, shrinkage reaching 81% after light, constant with increasing stretch ratio; in the presence of load, the greater the applied load, the smaller the shrinkage, 900% of the studies for the stretch ratio) (Fig. 4G). This strategy drives the development of research on light-dependent deformation. At the same time, a new well-stabilized LLCP with a wide range of intermediate phase temperatures and photosensitive properties, which contain azo esters by ring-opening translocation polymerization called PC11AE6, was synthesized [49]. Spontaneous orientation of PC11AE6, photo-induced deformation, and the relationship between light intensity, film thickness, and temperature were investigated. The highly ordered rigid azobenzene mesogen structure contributed to the flexibility of PC11AE6. Through 2D-wide angle x-ray diffraction characterization, it was observed that PC11AE6 spontaneously forms a uniform mesophase orientation in both low-angle and high-angle regions. The film possessed an anisotropic structure, which was smectic A phase at room temperature and in the bonded smectic C phase had an anisotropic structure above the phase transition temperature. The mesogens became the zigzag tilting in the lamella of smectic C phase, which showed the ability of PC11AE6 to spontaneously orient. Because the internal resistance of thick films was greater than that of thin films, films and fibers bent in the direction away from the light source, and thin films (10 μm/68°) bent more than thick films (20 μm/21°) under 365-nm UV irradiation. The higher the intensity of the radiated light, the more the film bent, as the higher photon energy drove more isomerization. The photo-induced deformation was performed in the coating A stage at a high temperature of 60 °C. The film was exposed to the light source for 5 s, and upon removal of the light source, it quickly recovered. This behavior was attributed to the instability of the cis structure at high temperatures. The results of the above investigations provide insight into the design of LLCP with a wide range of intermediate-phase temperatures. An LLCP with an azobenzene structure as a side chain was designed in 2021 (Fig. 4H) [50]. Under UV irradiation, the glass transition temperature on the surface of the LLCP film on the polymethyl methacrylate (PMMA) substrate was reduced to −39 °C, increasing viscous flow dynamics, “convex” shape, and channel cross-sectional area. Under 470-nm blue light radiation, the liquid slug moved away from the irradiated light source, but it passed through many complex pathways and could also perform operations such as liquid merging (Fig. 4I). The cross-sectional area of the channel is isosceles to achieve integrated operations such as movement, separation, and merging, which is difficult to ignore. Finally, this microfluidic chip was used for the first time in catalytic reactions and biological protein detection, opening the idea of portable testing.

Yu et al. [51] used a bottom-up 2-step synthesis and a single crystal-single crystal approach to fabricate large-sized platinum-based linear polymer crystals, in which ligand-dependent self-assembly was used to confirm that anthracene could achieve head-to-toe and head-to-tail formation by [4+4] cycloaddition, thereby forming the desired polymer crystals. Major investigations were carried out to follow its structural evolution during polymerization and depolymerization using x-ray diffraction techniques, and the depolymerization properties of polymer Pt-BA2DA slowly progressed with temperature. By single-crystal x-ray diffraction analysis, Pt-BA2DA single crystals were irradiated with white light at room temperature for 7 days to get Pt-BA2DA-i, which is 44% polymerized in the anthracene group, and longer irradiation time could not increase the degree. Therefore, a lower temperature of 265 K was used and 100% polymerization was obtained in 7 days for poly(Pt-BA2DA). The polymer depolymerization process is closely related to the heating temperature, and the 2 depolymerization processes require different energy barriers. Poly(Pt-BA2DA) to Pt-BA2DA-i has a lower exchange barrier, so the depolymerization rate is faster. To study the photodeformation behavior of the polymer, it was compounded with poly(vinylidene fluoride) (PVDF) to prepare thin films that bent toward the light source under light illumination. The synthetic strategy for preparing large Pt-based polymer crystals reported in this study has broad implications. Atom transfer radical polymerization was used to prepare polyacrylate with a flexible spacer, and azo-phenyl group as the main chain in the side chain of the polymer and cyclic gel permeation chromatography was used to prepare polymers of different molecular weights to be investigated (Fig.4J) [52]. They explored the photo-induced solid–liquid transition of polymers and photo-induced photomechanical response, mechanical properties, and healing ability of nitrogen-containing polymers. Since the polymer in this work is an entangled linear azide polymer, they are tougher and more mechanical and machineable compared to small-molecule polymers. The films exhibited photo-induced reversible bending behavior under UV illumination with or without stretching of the azobenzene intermediate at 90 °C to obtain aligned alignment. Photo-induced reversible solid–liquid exchange softens the freestanding films (photoisomerization causes the conversion of the light-emitting surface into a *cis*-rich state and the *cis* state into a viscous polymer melt) and bends more easily. Photo-induced reversible solid–liquid conversion enables the repair and reprocessing of azide polymer photoactuators with light at ambient temperature and in the absence of solvent. This polymer-entangled nitrogen-containing benzene polymer with photo-induced reversible solid–liquid conversion could help in the future design of other curable actuators. In 2022, a heterogeneous bead–spring model based on the photoreversible molecule azobenzene as the tailing group was developed. The kinetic relaxation behavior of this model in *cis*-*trans* isomerization is clearly described and analyzed in terms of photophysical calculations [53]. Furthermore, both models of *cis*-*trans* isomerism exhibited good photochromism and exhibited different states at room temperature (*trans*-hard solid, *cis*-soft solid). The tail-end relaxation time decreased with increasing *cis* content. However, at high temperatures, this conclusion is difficult to detect.

### LCPs with other structures

Photoinduced LCP materials with azobenzene photosensitive groups are attractive, are precisely and remotely operable in specific regions, and offer some opportunities for applications such as actuators. However, there are certain drawbacks to consider, notably the inherent thermodynamic instability of *cis*-azobenzene, which can undergo spontaneous conversion to *trans*-azobenzene. As a result, the transformation is not maintained for a long time even at room temperature, which limits the application of photo-deformable polymers to some extent. Therefore, in addition to the photosensitive group azobenzene, many other structures have been used to produce photodeformable polymers.

Some types of photosensitive groups can be used directly to construct LCEs with photosensitivity. A physically crosslinked photosensitive LCE (LCE-PM10PVPCm) was developed using α-cyanostilbene as an intermediate with different alkoxy tail lengths [54]. Characterization of monomer and polymer revealed that the higher the temperature, the stronger the hydrogen bond interactions of polymer chains. And the thermal stability of polymer proved by thermal decomposition experiments. With the increase of the alkoxy tail length, the *T_g_* decreased, while the LC phase was more stable. The uniaxially oriented LC elastic fibers prepared by the melt stretching method bend in the light emission direction under light irradiation because the polymer undergoes *cis*-*trans* isomerization when excited by UV light, the maximum bending angle of *m* = 4 is 113°, and the experiment also proves that more alkoxy groups are not favorable for a molecular motion to hinder isomerization.

**Fig. 5.**
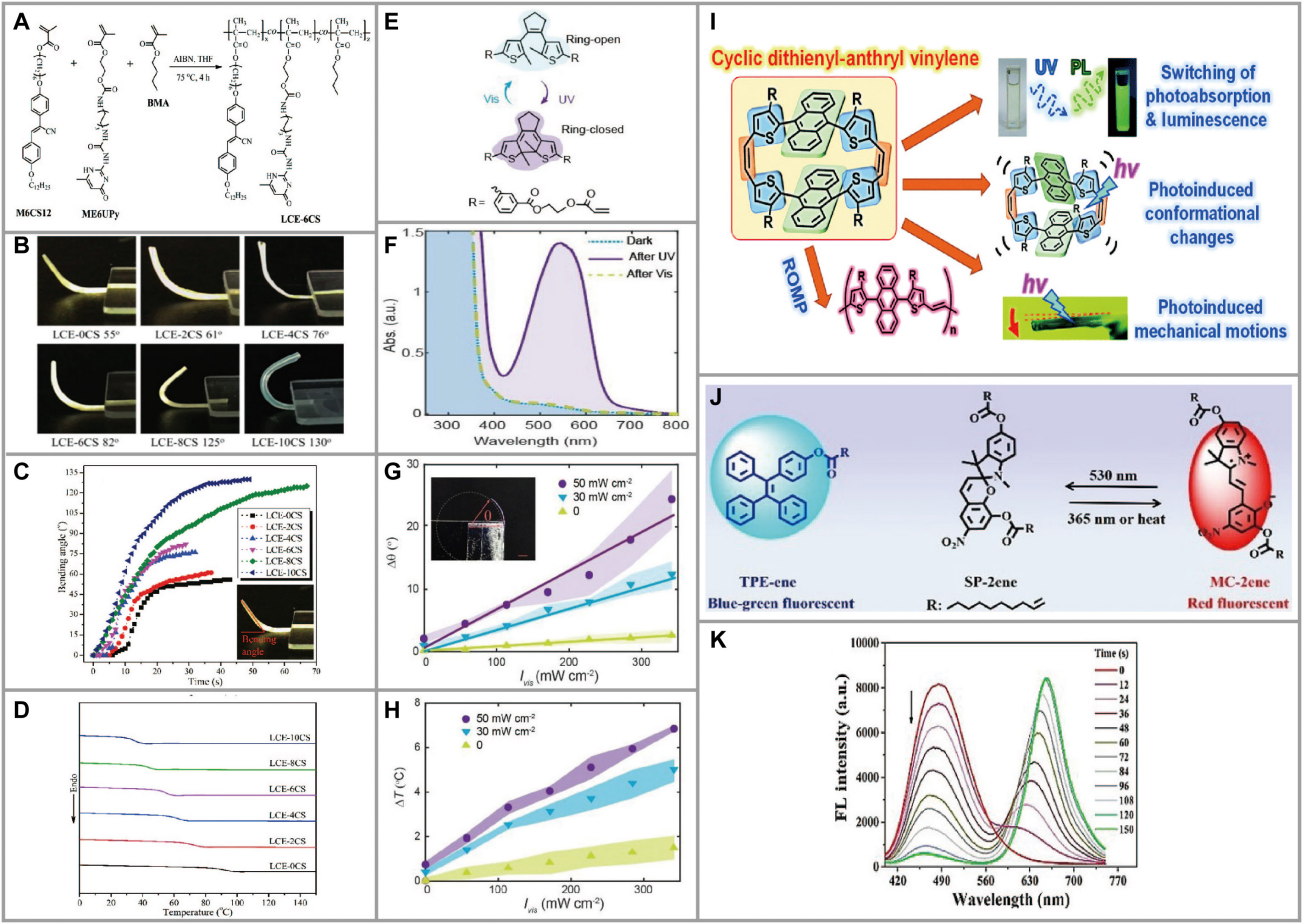
(A) Synthesis routes of LCE-6CS. (B) Pictures of LCE-mCS fibers, which has the maximum bending angle. (C) Bending angle change of LCE-mCS fibers upon irradiation time with UV light. (D) DSC curves of LCE-mCS fibers. Reproduced from [55] with permission from the Royal Society of Chemistry. (E) Chemical structures of the ring-open and ring-closed forms of the diarylethene crosslinker. (F) UV–Vis spectra of the DAE-LCN film before and after electrocyclization. (G) Bending angle change of the DAE-LCN strip upon irradiation with UV and visible light with different intensities. (H) Temperature change of the DAE-LCN strip upon irradiation with UV and visible light with different intensities. Reproduced from [57] with permission from the American Chemical Society. (I) Schematic of the multifunctional cyclic dithienyl-anthryl vinylene. Reproduced from [58] with permission from the Royal Society of Chemistry. (J) Chemical structure of TPE-ene and the reversible conversion between the open-form MC (red fluorescence) and the closed-form SP (nonfluorescence) of a functionalized spiropyran. (K) Time-dependent fluorescence spectra response of LCE1 film (λ_ex_ = 365 nm) with UV light. Reproduced from [59] with permission from Wiley-VCH.

*Cis*-α-cyanostilbene has good thermal stability. Regardless of the *cis*-*trans* conformation, they are not uniformly distributed in the radial direction of the fiber and can exhibit shape memory properties. Zhang et al. [55] synthesized a series of multi-hydrogen-bonded crosslinked α-cyanostilbene-based LCEs with different flexible spacer lengths (LCE-mCS, m = 0, 2, 4, 6, 8 and 10) (Fig. 5A and B). The issues of polymer thermal/liquid crystal properties, the effect of flexible spacer length on photodeformation behaviors, and the applications of LCE-mCS were investigated. The experimental analysis presented that *T*_g_ of all LCE-mCS decreased with increasing spacer length (Fig. 5C and D). Under UV irradiation, the uniaxially oriented LCE-mCS fibers bend along the fiber axis toward the light source, and both the bending speed and the bending angle increase with increasing spacer length. This is the result of the influence of *T*_g_. The maximum tilt angle of LCE-mCS reaches 130°. Compared with uniaxially oriented photochemically reactive LCE fibers with azobenzene, LCE-mCS shows great advantages in bending angle and bending speed. The deformation of LCE-mCS fibers is attributed to the *trans*-*cis* isomerization of α-cyanostilbene, and its photothermal effect and the plasticization effect of cis are greater than those of trans. In addition, the application of LCE-mCS in information encryption materials is explored, which provides a new idea for the preparation of new efficient photosensitive LCEs. Liao et al. [3] used poly(4-vinylpyridine) (P4VP) as the main chain and α-cyanostilbene derivative (Z-TCS) as the side chain, and both formed a series of supramolecular polymers of P4VP (Z-TCS)*x* in different molar ratios. This polymer undergoes photodeformation behavior under UV light irradiation, and the rate of bending increases with increasing *x*. The Z-TCS intermediate undergoes an order–disorder phase transition, and partial absorption of light energy by the α-cyanostilbene intermediate in the polymer for Z- and E-isomerization causes the P4VP backbone to contract, which together cause asymmetric contraction. At the same time, the *T*_g_ of the polymer decreases as the plasticizing effect of Z-TCS increases. *x* = 1.0 fibers bend to 90° in just 8 s, and the maximum bending angle of all P4VP(Z-TCS)*x* fibers is 135° or more. In addition, P4VP(Z-TCS)*x* fibers can be used with a photothermal effect for applications in information encryption and other smart manufacturing materials.

Diarylethene is one of classic photochromic chromophores [56]. The units of diarylethene undergo ring-closing and ring-opening via a 6π electrocyclization. LCN based on diarylethene (DAE-LCN) was designed [57]. The diarylethene unit and LCN were photopolymerized through 2 acrylate groups (Fig. 5E). The covalent bond between them ensures the stability of connections (Fig. 5F). The ring-closed diarylethene unit provided the large conjugated aromatic structure increasing the rigidity of the molecule. The bending angle varied for different UV intensities, and small bending is possible under a 550-nm lamp without using UV light (Fig. 5G and H). The increase of intensity made the diarylethene unit capture more light energy and thus the heat energy converted increases, which is macroscopically shown as a large bending deformation.

A complex maneuver was used to synthesize a cyclic, linear polymer with 9,10-dithiophenanthracene as the head group (Fig. 5I) [58]. This group can exhibit a unique conformation under UV, and the introduction of 3-hexylthiophene onto anthracene greatly inhibits the photodimerization of the anthracene group. These 2 polymers were prepared as polyethylene and polyamide films, respectively, and both exhibited good photodeformation behaviors. This leads to swelling mainly due to the distortion of the molecular structure of the thienyl-anthracenyl-thiophene group under light. Although there is some *cis-trans* structure that affects deformation, this type of isomerization is too rapid to produce a small effect.

Two fluorescence-tunable groups, tetraphenylene and spiropyran (SP), were used to bind to LCE (Fig. 5J) [59]. The photodeformation behavior of LCE is controlled by NIR initiation. Deformation and luminescence phenomena were studied by adjusting the presence of photothermal dye (YHD796)/tetraphenylethene (TPE)/SP. The group alternated between 530-nm visible light and UV light and found that the color of fluorescence changed between blue–green and red (Fig. 5K). Blue–green fluorescence is controlled by TPE, and red is controlled by merocyanine (MC). Irradiation with visible light for 90 s caused MC-SP to heterodimerize, during which excitation with UV radiation showed blue–green fluorescence. In addition, changing the MC structure of SP causes the fluorescence color to slowly change from blue–green to red under UV irradiation.

In addition to LC crosslinking, direct copolymerization can also achieve photodeformable behavior. Zhang et al. [60] synthesized a polyimide (azo-PI) polymer containing azobenzene as a photodeformable polymer material and explored its deformation behavior under unpolarized light. Since light-induced molecular alignment causes the azo-benzene network to expand or contract concerning polarization and ultimately conform to macroscopic deformation, it is necessary to induce molecular alignment by stretching and introducing flexible linker molecules into it. Thermally stretched azo-PI films were able to demonstrate their reversible bending behavior under alternating UV and visible light irradiation. In addition to this phenomenon that could occur in air, it could also occur in hot water (silicone oil) at 80 °C. It bent toward the light source within 8 s (12 s) after irradiation, reached a bending angle of 45°, and returned to its original position under green light. This showed that PI had good thermal stability and mechanical properties. This work motivated the design of promising actuators for azo PI for high-temperature applications. A polymer was formed with the hydrophilic monomer hydroxyethyl acrylate copolymerized with the monomer azobenzene, and another triple copolymer based on *N*-vinyl-2-pyrrolidone was formed [61]. The light response time of both copolymers was 30 s, which is higher than the light response time of other literature, and the recovery time of 110 min under white light irradiation was also slow. But the reason for their slow recovery was that this group used light intensity, solvent properties, and ambient temperature to influence the time faster or slower. The higher the light intensity, the shorter the recovery time.

He et al. [62] showed for the first time a functional polymer single crystal obtained by self-inoculation with polyethylene glycol (PEG) using azobenzene as a capping group. It was confirmed that the *trans*-*cis* isomerization of azobenzene still occurs well in light even with a single crystal structure. mPEG-Azo flaked rapidly isomerized as in a solution. This indicates that the environment around the azo-phenyl groups attached to single crystals is relatively loose. Although the synthesis of these polymer single crystals is relatively simple, they cannot have a high degree of freedom compared to polymer materials, such as bending and twisting actions.

## Photodeformable polymer materials of Hydrogels

**Fig. 6.**
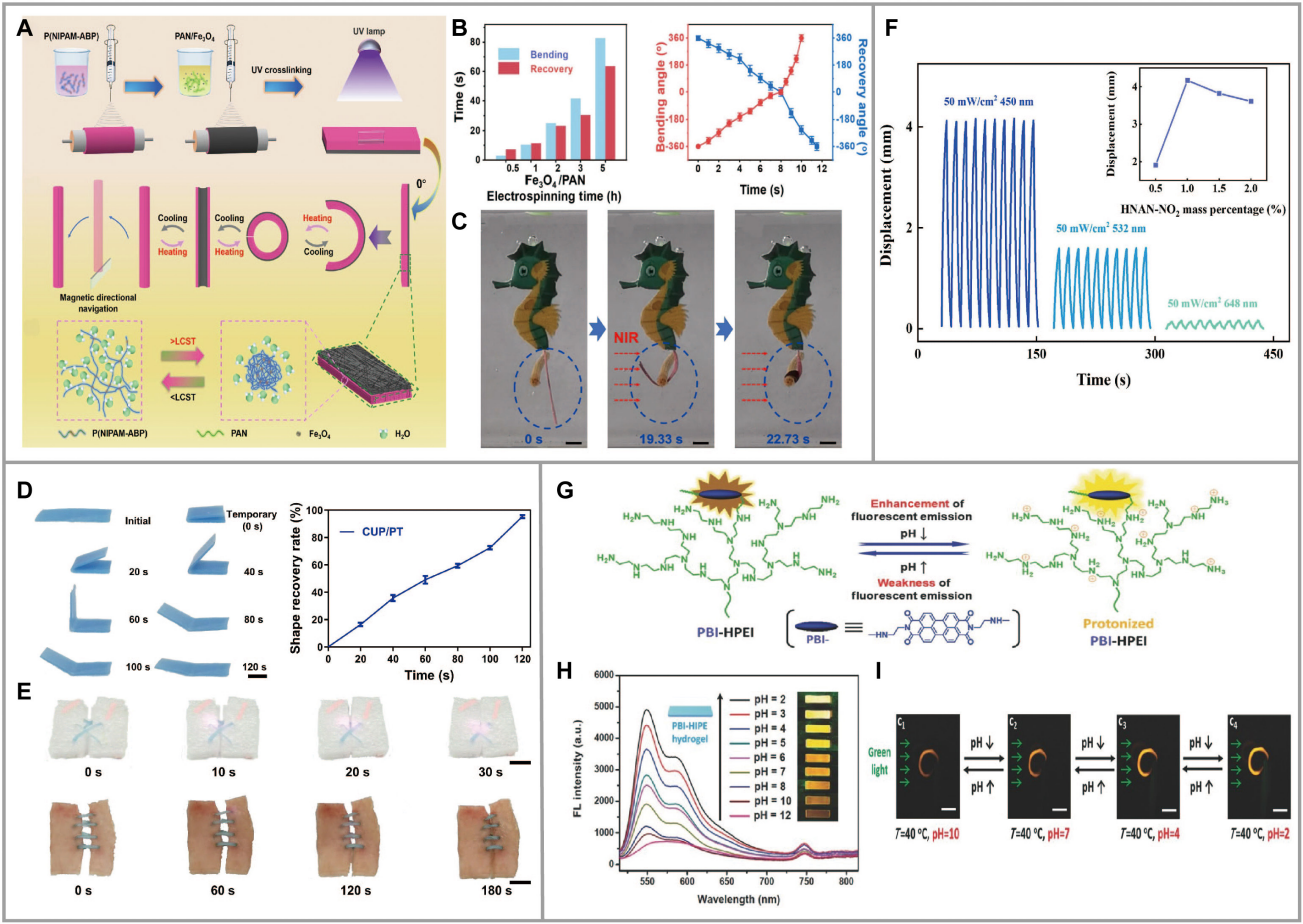
(A) Preparation process of FPP hydrogel actuator. (B) Relationship between the electrospinning time of Fe_3_O_4_/PAN and the response time of the deformation recovery time. (C) Bending speed of the actuator under NIR and the recovery speed in cold water. Reproduced from [65] with permission from Elsevier. (D) Photographs of shape recovery of CUP/PT composite under irradiation of NIR-II light, and its ratio over time in the bending mode. (E) Photographs showing the self-knot behavior of CUP/PT composite using foams and pigskins as in vitro wound model, respectively. Reproduced from [66] with permission from Elsevier. (F) Light-induced displacement of the 1 wt% HNAN-NO_2_/P(VDF-TrFE) composite film with 3 types of wavelength light illumination. Reproduced from [67] with permission from Elsevier. (G) Chemical structure of PBI-HPEI (perylene bisimide-functionalized hyperbranched polyethylenimine) hydrogel, which has pH-responsive fluorescent color-changing principle. (H) Fluorescence emission spectra of PBI-HPEI hydrogel with changes of pH (λ_ex_ = 532 nm). (I) Schematic of fluorescent color changing after shape deformation with pH. Reproduced from [68] with permission from Wiley-VCH.

Hydrogel structure is a kind of smart material, which has been used in drug delivery, tissue engineering scaffold, medical cosmetic material, etc. According to the difference in their crosslinking methods, physical synthetic hydrogels are crosslinked by physical interactions. While hydrogen bondings, van der Waal forces, and pseudo-chain entanglement between different polymer chains are reversible, chemically synthetic hydrogels with solid chemical bonding crosslinking are, in contrast, irreversible. Changing hydrogels from 2D planar structures to 3D structures can be achieved by changes in the bilayer structure, changes in thickness, and changes in plane. In general, the stimulus sources that affect the degree of hydrogel change are pH and temperature [63,64], and light has great potential of attracting attention as a green and clean energy source. Combining electrostatic spinning technology with composite hydrogels improved the mechanical properties, responsiveness, etc. of the hydrogel fabrics, which improved how the hydrogel actuators performed complex programmed movements (Fig. 6A to C) [65]. They doped Fe_3_O_4_ nanoparticles in polyacrylonitrile (PAN) solution, 4-acryloylbenzophenone and *N*-isopropylacrylamide copolymer P(NIPAM-ABP) were spun separately, and crosslinked polymerization was carried out by UV to obtain Fe_3_O_4_/PAN-P(NIPAM-ABP) composite hydrogels (FPP hydrogel). These hydrogels had no temperature-responsive properties due to the incorporation of a PAN magnetically responsive layer. Fe_3_O_4_ increased the binding force between nanoparticles and PAN electrostatic gravitational forces and van der Waals forces. With the increase of Fe_3_O_4_ content, its mechanical strength first increased and then decreased. The most suitable doping amount was Fe_3_O_4_/PAN = 2.5:1, and the strength of composite hydrogel reached 59.250 MPa. Not only that, but this hydrogel also achieved a transition from −360° to +360° in 9.7 and 11.6 s for actuator recovery.

## Photodeformable polymer materials of shape memory polymers

Shape memory polymer materials (SMPs) are another type of smart material that is limited by programming complexity beyond hydrogels and LC materials, requiring an externally applied stress for programming. Unidirectional SMPs result from a change in the amorphous phase transition of their polymers, and the temperature of this transition is the glass transition temperature, *T*_g_, which is approached with a subsequent increase in the stiffness of the material, e.g., SMCs are composites made from a unidirectional SMP and an elastic polymer or 2 different unidirectional SMPs. At different temperatures, the SMP and elastic polymer dominate differently, with high-temperature SMP being more dominant, causing stress in the deformed elastic polymer. Huang et al. [66] prepared a NIR-II light-responsive cuprorevit/poly (d,l-lactide-co-trimethylene carbonate) (CUP/PT) composite material. Under low power NIR-II radiation of 0.4 W cm^−1^, the folded film could recover the flat shape in 120 s and had good repeatability and stability (Fig. 6D). The composite sutured 2 separated foam or pigskin connections in 30 or 180 s, providing a promising application for deep tissue wound healing (Fig. 6E). The synthesis of polyvinylidene difluoride/Schiff base composites, which demonstrated significant photodeformable behavior under visible light (blue, green, and red), was obtained. This behavior was attributed to the macroscopic deformation of the Schiff base, resulting from intramolecular proton transfer and bond rotation. Notably, the composites exhibited an induced lattice transition when illuminated (Fig. 6F) [67].

## Photodeformable polymer materials of Carbon-based materials

Carbon-based materials such as carbon nanotubes, graphene, and amorphous carbon have been considered highly efficient photothermal materials with low cost and good photothermal conversion efficiency. Carbon-based materials can be used as special functional layers, doped molecules, and nano-fillers to improve the mechanical properties of various devices. Li and Wang [68] developed the first photo-wet responsive all-carbon actuator, which consisted of a bilayer material consisting of hydrophilic/hydrophobic graphene oxide (GO) layers vacuum deposited on oriented carbon nanotubes, which was achieved by NIR light with deformation in only 0.08 to 0.24 s. PNIPAAM remains expanded when the temperature is not yet above the critical solution temperature and changes to the contracted state when the temperature rises above the lower critical solution temperature. To overcome the low thermal conductivity problem of the common PNIPAAM actuator, doping with carbon-based materials was performed. An actuator that alters the fluorescent color change and can be programmed with complex shapes by mixing GO-PNIPAAM composites with pH-responsive perylene bisimide functionalized hyperbranched polyethylene to obtain composites was reported (Fig. 6G and H) [69]. Actuator deformation and fluorescence color were changed to different degrees under the influence of green light, temperature, and pH (Fig. 6I).

## Application of photodeformable polymer materials

Due to their properties such as flexible degrees of freedom and differences in energy absorption, LCPs can achieve a wide range of applications that cannot be achieved by other structural types. Not only that, this rational structure of theirs is also applied in devices with high functionality and precision of light control.

### Biomimicry

The natural world has produced sophisticated, complex, and intelligent living systems that have served as inspiration for material design. Flexible LCP materials offer adaptive advantages in mimicking soft biological structures, and the combination of photoresponsive driving enables the design of intelligent microsystems that mimic biological behavior.

**Fig. 7.**
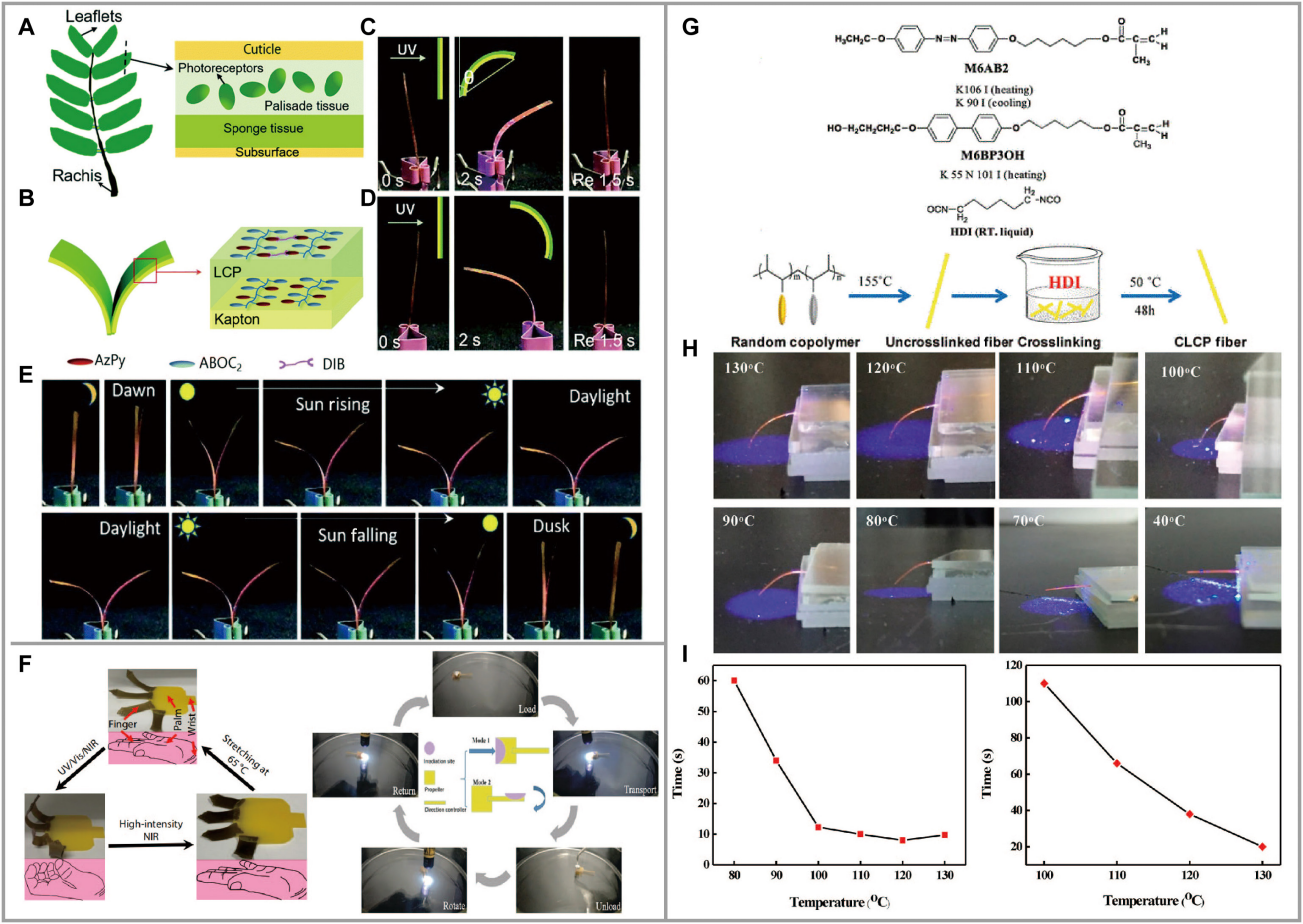
(A) Scheme of silk tree leaves with symmetrical leaflets along the rachis. (B) Scheme of light-triggered artificial leaves designed in this work. (C) Photomechanical behaviors of the LCP–Kapton bimorph composite films. UV light was shined on the LCP side from the left. From left to right: UV off, UV on for 2 s, recovery for 1.5 s. (D) UV light shone on the Kapton side from the left. Inset is a scheme for the bending behavior of bilayer film (green: LCP layer; yellow: Kapton layer). (E) Opening and closing movements of the artificial leaves. Reproduced from [70] with permission from Spring Link. (F) Biomimicry for the opening of fingers and the swinging forward of swimmers. Reproduced from [35] with permission from Wiley-VCH. Reproduced from [71] with permission from Wiley-VCH. (G) Chemical structures and schematic illustration of fabrication process of CLCP fibers. (H) Photographs of maximum bending of the AZ-CLCP fibers at different temperatures. (I) Relationship between the temperature and photoinduced bending motion of AZ-CLCPs fibers. Reproduced from [73] with permission from the American Chemical Society.

Bimorph composites fabricated using polyamide (Kapton) substrates and LCP layers copolymerized with 2 monomers M6ABOC2/M6AzPy, and bionic sleep of *Albizia julibrissin* leaves were explored (Fig. 7A and B) [70]. Chemically crosslinked films were found to reach photo-equilibrium within 5 s. Under UV irradiation, the maximum displacement angle was reached in 2 s and the films bent toward the Kapton layer regardless of the direction of irradiation (Fig. 7C and D). In the theoretical model, 2 relationships were observed while keeping the Kapton thickness constant. Firstly, the light driving force was found to be proportional to the radiated light intensity. Second, the displacement angle was also found to be proportional to the radiated light intensity. It was further observed that as the LCP layer thickness increased, both the light driving force and displacement angle also increased. In addition, under gradient UV radiation, artificial leaves showed circadian rhythmic motion according to the light changed from “dawn” to “dusk” (Fig. 7E). This intelligent light sensing and motion execution system pave the way to mimic the fine motor control system in natural plants.

Nowadays, many photoresponsive LCPs are used to design bionic functional applications. When driven by light, they regulate the circadian rhythm of plants, the opening of fingers, the movement of caterpillars, the blooming of flowers, the swinging forward of swimmers, and other shape changes or movements (Fig. 7F) [33,41,59,71,72]. The results of these studies have provided creative inspiration for functionalized applications of light-responsive LCPs.

### Encryption storage materials with synergistic photothermal effects

Photothermally responsive polymer materials are heated locally by light control. If the temperature of the heated region is higher than the *T*_LC-iso_, the LC radicals randomly lose their previous orientation direction, thereby restoring the polymer chains to a random curl conformation. During the phase transition, the polymer undergoes contraction parallel to the initial direction of the LC and expansion in the perpendicular direction. Once cooled below the transition temperature, it spontaneously reverts back to its original shape due to self-alignment.

In 2017, Yu et al. [73] fabricated LC post-crosslinked random copolymers (AZ-CLCPs) with azobenzene as a side chain (Fig. 7G). The effect of copolymers containing different AZ concentrations and crosslinking densities on the photoresponsive behavior was investigated. The amorphous LC copolymer became soft when the temperature reached above 80 °C, and it rapidly melt under UV irradiation. The photo-softening and melting behavior of the amorphous LC copolymer were attributed to the photo-induced phase transition from the LC phase to isotropic phase when the temperature exceeded *T*_g_. However, after crosslinking, the AZ-CLCP fibers exhibited highly ordered interfacial morphology, and under the combined influence of light and temperature, they could achieve light-induced bending. Below the *T*_g_ temperature, the chain segments were frozen and both crosslinked and uncrosslinked fibers remained unchanged. When the temperature reached 80 °C, the photodeformation behavior appeared rapidly (Fig. 7H). The bending rate of fibers increased linearly with increasing temperature. At higher temperatures, the recovery rate of fiber surface expansion induced by AZ photoisomerization became faster (Fig. 7I). Below *T*_LC-iso_, fiber programming could be achieved by controlling the temperature to shape the fibers and achieve an encrypted effect.

**Fig. 8.**
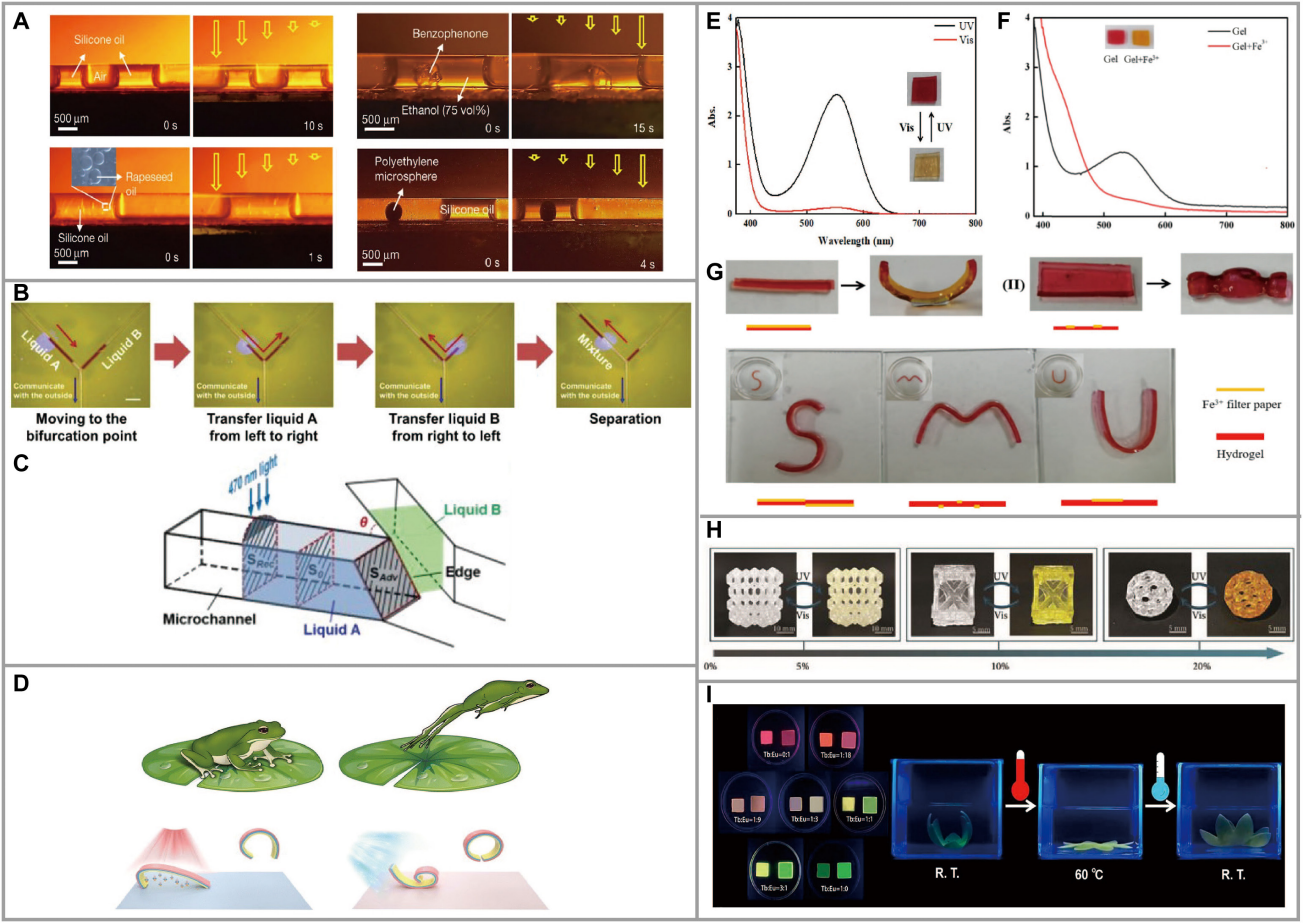
(A) Lateral photographs showing light-induced motion of a biphase fluid. All photographs were taken through an optical filter that removed light with wavelengths below 530 nm. Reproduced from [45] with permission from the American Chemical Society. (B) Photographs showing the fusion of 2 ethanol slugs (dyed red) in junction channels. (C) Simulation of the fusion and separation of 2 slugs during irradiation. The crossing section area of the microchannel is denoted by S_0_. The angle between the 2 branch channels is denoted by θ. Reproduced from [50] with permission from Wiley-VCH. (D) Schematic illustration of frog-like jumping actuation. Reproduced from [76] with permission from the Royal Society of Chemistry. (E) The UV–Vis absorption spectrum of the hydrogel under UV and white light. (F) The UV–Vis absorption spectrum of the hydrogel containing Fe^3+^. (G) Deformation behavior of the hydrogel triggered by Fe^3+^. Reproduced from [77] with permission from Elsevier. (H) Photoresponsive pictures of the printed hollow 3D structures containing different mass fractions of TrPEF_2_-MA. Reproduced from [81] with permission from the American Association for the Advancement of Science. (I) Photos of fluorescent hydrogels doped with different molar ratios of Tb^3+^/Eu^3+^ mixed solutions before (right) and after (left, heated in water for 5 min at 60 °C) heating. Photographs of 3D-printed hydrogel-based artificial flower. Reproduced from [82] with permission from Elsevier.

A variety of photothermal reagents capable of forming composites with LCPs have been reported, including carbon nanotubes, graphene, metal nanoparticles, organic molecular dyes, conjugated polymers, and polydopamine. All of them can absorb light in a specific wavelength range, convert light energy into heat energy, and release heat into the polymer matrix, thereby driving shape change. Zhang et al. [71] fabricated an LC mixture containing azobenzene mixed with GO doped into PU to obtain shape memory PU (SMPU) films. This composite film has excellent response properties to UV–visible (Vis)–NIR, and bending behavior occurs due to various factors when irradiated by different light sources. The LC composite was composed of different ratios of 5CAZ/5CB, and experiments showed that the content of 5CAZ affected the photodeformation response under UV, while the corresponding GO played an important role under NIR. 5CAZ/5CB/GO (3 wt‰)/SMPU films could recover bending by high-intensity NIR light. The film achieves a continuous shape change through continuous thermal stretching and light irradiation. The biomimetic bending and opening of a finger could be achieved reversibly through a combination of continuous thermal stretching and light irradiation (Fig. 8F). When GO was introduced into the 5CAZ/5CB/SMPU thin film, a parameter of bending degree (*F* = 0.31) of the elongated 5CAZ/5CB/GO (3wt%)/SMPU film significantly increased after visible light exposure. During the visible light exposure process, the surface temperature of the stretched 5CAZ/5CB/GO (3 wt%)/SMPU film rose to 45.1 °C, higher than *T*_i_ (39.5 °C). This indicated that the visible light-induced bending deformation of the elongated 5CAZ/5CB/GO (3wt%)/SMPU film was attributed to the *cis-trans* photoisomerization of 5CAZ, while the photothermal-induced LC-to-I phase transition was originated from the photothermal effect of GO. However, this doping is doped with many things that affect its performance and the doping limit is unknown.

### Microfluidic light-controlled transport

Microactuators are commonly used in biomedicine, chemical analysis, and other fields and have potential application opportunities in bionic blood vessels, minimally invasive surgery, etc. The use of light to precisely control the advance, retreat, merging, and separation of fluids is now the pursuit of microfluidic technology. In addition, the microfluidic system can realize noncontact control of the fluid. The first article on LLCP integrated TMAs for optically driven manipulation of liquids was published in 2016 [45]. In contrast to previous reports that could only manipulate specific liquids, this work demonstrates that each type of TMA (straight, Y-shaped, serpentine, helical) could manipulate liquids ranging from polar to nonpolar, including silicone oil, hexane, ethyl acetate, ethanol, and water (Fig. 8A). Furthermore, it has potential applications in the field of biology. The research team also demonstrated the optically driven manipulation of commonly used biological liquids such as bovine serum albumin solution, phosphate buffer solution, cell culture media, and cell suspension. This simple TMA greatly simplifies the device for microfluidics and provides significant support in the field of microactuator technology. Furthermore, this work also breaks through linear motion within the plane, as the droplets could climb slopes at a speed of 0.05 mm s^−1^ with an inclination angle of 17°, providing assistance in the design of microfluidic reactors. Yu et al. [50] have mostly reported on microfluidic technology, which mainly used photodeformable LLCPs to fabricate the micropipe and PMMA as the substrate to achieve this fluid transport. Among them, the separation and merging of the liquid was mainly affected by the cross-sectional area, and the length of the radiant side was reduced so that the surface tension on both sides was unequal, resulting in fluid movement. Through research and exploration, the most suitable cross-sectional area should be considered as an isosceles trapezoid (Fig. 8B and C). However, such dynamic LLCPs can only be driven under UV lamps, whose deep penetration greatly hinders their biological application.

### Actuators

Because of the current problems of how to eliminate light stimulation operation, configuration limitations, etc., it is imperative to improve the directionality of movement accuracy and control soft actuators and fast response mode since actuators guided by UV light have precise scanning direction constraints. Therefore, it is important to adopt a light source that can replace UV light and thus guide actuators with multiple motion patterns. In this respect, NIR light has an advantage due to its fewer side effects and less toxicity in biological tissues. NIR light has a high selectivity in the wavelength region and low light penetration. This allows NIR light to be effectively separated from other wavelengths, avoiding interference and false triggering. This selectivity is critical in the precise control and manipulation of photodeformable materials [74]. Besides, NIR photodeformable materials typically have good repeatability and long lifetime.

Organic absorbers in the NIR include compounds such as benzobisthiadiazoles, dinuclear ruthenium complexes of 1,2-dicarbohydrazide ligands, and metal dithiones, but the synthesis of these materials is more complex. Based on this, Chen et al. prepared a synthetically simple NIR-absorbing diol and applied it to the crosslinked elastic polymer of PU to obtain PU-T [75]. To compare the effect of the structure of the diol on the deformation behavior, a diol-free polymer PU-BL was prepared. Under the irradiation of NIR light at 808 nm, the expanded PU-T restored its initial state within 60 s. In addition, it was able to lift 160 times its weight, indicating that the material with this structure has good photodeformation behavior and photothermal stability and that it has a wide range of absorption capabilities (700 to 900 nm).

A bidirectional switch, a bidirectional walker, and a visible/infrared triple wavelength modulated walking robot, all of which can effectively control the gait and deformation of the actuator, providing ideas for multistimulus responsive soft actuators [30]. The actuators exhibit strong macroscopic motion and fast response speed and can be easily operated using stimuli. Zhou et al. [76] made a dual-responsive jumping frog, providing a new design strategy for actuators (Fig. 8D).

Designing simple molecules and precisely modulating the molecular structure is key to the development of NIR photodeformable materials for future applications. Modulation of actuators using NIR and visible light enables fast response over a wide range, while the multistep response increases the applicability of flexible actuators. Combining NIR photodeformable materials with other functional materials can realize multifunctional integration. This multifunctional integration will promote the expansion of the application of NIR photodeformable materials. At the same time, by regulating the optical, electrical, magnetic, and other properties of the materials, it has photoelectric conversion, sensing, manipulation, and memory effects and other characteristics. It will open up new possibilities for implementing more complex functional systems.

### Dual effect of light response distortion and color change

Inspired by chameleons and color-changing insects in nature, we can change the appearance and color of materials through multiple stimuli. In the LC materials, the azo phenyl group is of wide interest as it can be isomerized upon light exposure, and in addition to this group, SP is also a potential isomerization group, which is also responsible for color change. In general, the isomerization group induces a color response to temperature, pH, light, and stress. A hydrogel composed of *N*-isopropylacrylamide (NIPAAm), acryloyl 6-aminohexanoic acid (A6ACA), and spiro-acrylamide (SP) derivatives was developed [77]. This hydrogel can change its color and deformation with the presence of Fe^3+^. In the absence of Fe^3+^, the incorporation of Fe^3+^ caused anisotropic swelling in selected regions of the hydrogel, leading to macroscopic deformation of the hydrogel (Fig. 8E to G). Not only that, the absorption peak at 540 nm of red hydrogel after UV irradiation disappeared to yellow color after soaking by Fe^3+^. This provides a new idea for the dual response of color-changing deformation.

Our group explored a new isomerization group, the tristyryl group, which has the advantages of simple structure, convenient synthesis, longer cycles, and lower cost compared to other groups since 2016 [78–80]. We applied it to the polymer material PMMA to form thin films to explore the properties of data encryption, rewritability, and others. Based on this, digital light-responsive 3D structures are made of methyl methacrylate containing triphenylene (TrPEF_2_-MA) combined with 3D printing technology, which is expected to combine shape memory materials and LC materials to achieve the dual effect of color change and deformation (Fig. 8H) [81]. The hydrogel is also achieving this dual effect through 3D printing as shown in Fig. 8I [82]. A predesigned hydrogel model was printed by an LD-001 3D printer. The slice thickness of each layer was 50 μm during the printing. The exposure time of each layer was set to 3 s, except for the first layer, which was set to approximately 180 s. The obtained structure (PANT hydrogels) was rinsed in a sonicator bath for 1 min to remove the monomers/PI residues, and it needed to be stored in a sealed environment. The PANT hydrogels were immersed in a 0.1 M solution of Eu(NO_3_)_3_ and Tb(NO_3_)_3_ for 15 min to get the Eu/Tb-PANT hydrogels. With different molar ratios of Tb^3+^/Eu^3+^, they have dynamic fluorescent colors under UV@254 nm. Based on this, dual effect was realized.

### Other Applications

In addition to these bionic, photo-thermal synergistic encrypted storage, microfluidic, and color-changing deformation dual-action applications, LCPs can also be applied to sensors, autonomous healing, and drug transport [40,83,84].

Self-healing is a way to delay the life and life cycle of materials and improve their reliability. Smart materials with this capability have far-reaching implications for engineering manufacturing, medical implants, and other applications where damage to materials is irreversible and prohibitively expensive. The article by Patrick et al. [84] reviewed 3 different mechanisms of self-healing at 3 different levels: At the molecular level, broken bonds are repaired by supramolecular interactions such as ion pairing; at the microscopic scale, microfractures can be bridged by adding effective additives or a mixture of repair agents and catalysts; and even more destructive, only vascular network delivery methods are used to achieve the healing function. Due to the demanding environmental requirements in terms of pH, temperature, humidity, and oxygen concentration, it is a significant challenge to improve the self-healing properties of materials. A simple 2-step method to synthesize photomechanical elastomers (PMEs) for optical drivers was presented [85]. Three different azobenzenes were used to produce the PME break film. Azobenzene molecules were covalently linked to the PU backbone in 2 phases and crosslinked by hydrogen bonding. The PME has unique autonomous self-healing properties, which exhibited a maximum absorption peak at 385 nm when measured in tetrahydrofuran. However, under UV irradiation, the absorption peak gradually decreased, while the absorption value in the visible region increased. After 30 s of irradiation, the curve returned to its original state. A solid PME film under UV irradiation had a complete bending motion time of 50 s. The highly dynamic hydrogen bonding conducted noncovalent crosslinking and formed a network that enabled autonomous self-healing. After cutting the arbitrarily aligned film into 2 pieces, the newly cut edges were exposed to room temperature for 5 min. Remarkably, the healed film exhibited the ability to stretch without breaking, reaching a strain of γ = 100%. Then, this image was applied to a light-driven soft robot gripper.

When the porous LCN acting as a substrate is stimulated, the fluid lubricant is preprogrammed to acquire fluid secretion sites and the capillary bridges are programmed for ligand adhesion, enabling self-regulated drug transport or chemical reagent release in response to stimulation. Under UV light irradiation, the secreted sensitive dye reagent is visualized by pH and its secretion can be detected by UV–Vis absorbance applied to the release of medical drugs.

### Summary and Outlook

Photodeformable polymer materials have obtained an increasing interest as an emerging field of research, due to their advantages of cleanliness, remoteness, tunable intensity and wavelength, and nondestructiveness under light excitation. In this review, we have summarized the classification of photodeformable polymer materials, from molecular design preparation to applications of photodeformable materials in various challenging fields. Additionally, we also described the challenges of their current development. For the photodeformable polymer materials, LCPs are regarded as the largest class, which have excellent reversibility and accuracy under light stimuli. Besides, other photodeformable polymer systems including hydrogels, SMC polymers, and carbon-based materials are also summarized.

In the past years, the development of photodeformable polymer materials has undergone remarkable advancement, particularly in the development based on the molecular design, group selection, and applications. However, there are still some concerning limitations impeding the achievement of high-performance photodeformable polymer materials. First, the kinds of photo-responsive substances that have been used in photodeformable polymer materials are relatively few. Apart from the widely studied azobenzene molecules, stilbene, cyclic photosensitive groups, transition metal complexes, conjugated polymers, and other groups can be applied in photodeformable polymer materials in the future, which is helpful to improve maneuverability and beneficial to enhance service lifetime for applications. Subsequently, most photodeformable polymer materials only respond to UV light, which limits their applications. By combining or doping substitutional groups that respond to visible or NIR light, it is possible to expand the range of dynamic modulation of light irradiation. Then, the stability of photodeformable polymer materials still needs to be strengthened to circumvent the light absorption dilemma. The choice of various types of crosslinking agents (photosensitive crosslinking agents, thermal crosslinking agents, chemical crosslinking agents, ionic crosslinking agents, and so on) plays a crucial role in crosslinking process. In terms of crosslinking mechanism, these crosslinkers vary in nature, which can achieve a selectively crosslinking process to allow for flexible shape morphing. In addition, it was found that not only photodeformable polymers are found in the form of film or fiber but also the absorption peaks of light sources are similar to the changing photosensitive groups, which limits the preparation of the actuator and makes it difficult to commercialize. To develop more high-performance and diverse applications, the study of photodeformable polymer materials could move toward materials for multiple stimulus response integration and robots with self-adjusting behaviors depending on environmental conditions. At the same time, developing some new applications, for instance, microtechnology, nanotechnology, photonics, and sensors, is also indispensable for future development. We hope that this review could provide new directions to solve problems and have further development in the field of photodeformable polymer materials.
